# Differential Binding of Carbapenems with the AdeABC Efflux Pump and Modulation of the Expression of AdeB Linked to Novel Mutations within Two-Component System AdeRS in Carbapenem-Resistant Acinetobacter baumannii

**DOI:** 10.1128/msystems.00217-22

**Published:** 2022-06-23

**Authors:** Subhasree Roy, Vivek Junghare, Shanta Dutta, Saugata Hazra, Sulagna Basu

**Affiliations:** a Division of Bacteriology, ICMR-National Institute of Cholera and Enteric Diseases, Kolkata, India; b Department of Bioscience and Bioengineering, Indian Institute of Technology Roorkee (IITR), Roorkee, Uttarakhand, India; c Centre for Nanotechnology, Indian Institute of Technology Roorkee (IITR), Roorkee, Uttarakhand, India; Marquette University

**Keywords:** Acinetobacter baumannii, AdeABC, AdeRS, PAßN, 3D modeling, molecular docking, antimicrobial resistance, carbapenem resistance, neonatal sepsis, India

## Abstract

Resistance-nodulation-division-type efflux system AdeABC plays an important role in carbapenem resistance among Acinetobacter baumannii. However, a knowledge gap is observed regarding the role of its regulator AdeRS in carbapenem-resistant A. baumannii (CRAB). This study effectively combines microbiological analysis with an *in-silico* structural approach to understand the contribution of AdeRS among CRAB (*n* = 38). Additionally, molecular docking was performed for the first time to study the interaction of FDA-approved carbapenems and pump inhibitor PAβN with the open and closed structure of AdeB at the three binding sites (periplasmic, proximal, distal). It was observed that open conformation of AdeB facilitates the binding of carbapenems and PAβN at entrance and proximal sites compared to the closed conformation. PAβN was found to block carbapenem interacting residues in AdeB, establishing its role as a competitive inhibitor of AdeB substrates. Overexpression of AdeABC was detected by q-RT-PCR among 29% of CRABs, and several mutations within AdeS (GLY186VAL, SER188PHE, GLU121LYS, VAL255ILE) and AdeR (VAL120ILE, ALA136VAL) were detected by sequencing. The sequence and structure-based study of AdeRS was performed to analyze the probable effect of these mutations on regulation of the two-component system (TCS), especially, utilizing its three-dimensional structure. AdeS mutations inhibited the transfer of a phosphate group to AdeR, preventing the binding of AdeR to the intercistronic region, leading to overexpression of AdeABC. The elucidation of the role of mutations in AdeRS improves our understanding of TCS-based regulation. Identification of the key residues of AdeB interacting with carbapenems and PAβN may help in future designing of novel inhibitors.

**IMPORTANCE** AdeABC is an important efflux pump in A. baumannii that plays a role in resistance toward different antibiotics including the “last resort” antibiotic, carbapenem. This pump is regulated by a two-component system, AdeRS. To understand the binding of carbapenems with AdeABC and pump inhibition by PAβN, we analyzed for the first time the possible atomic level interactions of carbapenems and PAβN with AdeB. In the current study, AdeRS-associated novel mutations in clinical A. baumannii are reported for the first time, and a sequence-structure based *in-silico* approach was used to interpret their role in AdeABC overexpression, leading to carbapenem resistance. None of the previous studies had undertaken both these aspects simultaneously. This study analyzes the open and closed conformation of AdeB, their binding with carbapenems, and key residues involved in it. This helps in visualizing the plausible atomic level causes of pump inhibition driving the discovery of novel inhibitors.

## INTRODUCTION

Acinetobacter baumannii is among the critical pathogens in the WHO priority pathogen list (1). This inclusion is not without reason, as this organism is a significant clinical threat due to its ability to resist most antibiotics and persist in the nosocomial environment. The rates of multidrug resistance in A. baumannii are nearly four times higher than other Gram-negative bacilli (GNB) (2). The rapid global dissemination of A. baumannii resistant to carbapenems represents a significant clinical threat (3–6). It is considered that resistance against carbapenems is, in itself, sufficient to define an isolate of A. baumannii as highly resistant. Reports of outbreaks due to carbapenem-resistant A. baumannii (CRAB) in ICUs (intensive care units) and NICUs (neonatal intensive care units) have already been published (7–10). In India, the rate of infection due to CRAB is also reported to be high (11–15). It is a major cause of neonatal sepsis in developing countries (16–18).

Carbapenem resistance in Acinetobacter is primarily due to the production of carbapenemases (metallo-β-lactamases, oxacillinases) (4, 8, 15). Several metallo-β-lactamases (MBLs) have been reported in A. baumannii such as IMP (imipenemases), VIM (Verona integron-encoded MBL), SPM (Sao Paolo MBL), SIM (Seoul imipenemase), GIM (imipenemase from Germany), and NDM (New Delhi MBL) (4). OXA-23, OXA-24/40, OXA-58, OXA-143, and OXA-235 are examples of oxacillinases that are detected in A. baumannii and are able to hydrolyze carbapenems (4). Unlike the transmissible mechanisms of resistance nonenzymatic mechanisms such as efflux pumps, loss of outer membrane protein (OMP) and alteration of penicillin-binding protein remain unexplored in clinical microbiology laboratories but can cause treatment failure. Although there are several resistance mechanisms that specifically target a particular antibiotic group, broad substrate specificity is displayed by efflux pumps to expel unrelated chemical compounds with a wide range of chemistry (19). To date, three resistance-nodulation-division (RND)-type efflux pumps have been fully characterized in A. baumannii: AdeABC, AdeFGH, and AdeIJK. Apart from these three pumps, a further five “as-yet-uncharacterized” RND pumps have also been described in A. baumannii (20). These five pumps among A. baumannii genomes were detected using an *in-silico* method from the NCBI database. RND-type efflux pumps in GNB have great clinical significance.

RND efflux pumps are tripartite systems, driven by proton motive force. The pumps usually consist of a transporter protein located at the inner membrane. This transporter protein interacts with a membrane fusion protein (periplasmic) as well as with an outer membrane protein channel to permit the export of drug molecules across membranes. Among the three fully characterized efflux pumps, AdeABC was first characterized. It is similar to other RND pumps with a periplasmic protein (AdeA), inner membrane protein (AdeB), and outer membrane-associated protein (AdeC) (21). Su et al. analyzed the cryo-electron microscopic structure of the AdeABC pump and showed a plausible pathway for multidrug extrusion of antibiotics of different groups including a carbapenem (imipenem) (22). They elucidated the binding of different drugs to the periplasmic, proximal, and distal sites of the AdeB and suggested a mechanism for energy coupling that powers up this membrane protein (AdeB) to export antibiotics from bacterial cells. In a separate study, *adeB* was inactivated by plasmid insertion and mutants for the *adeB* gene showed a 4- to 6-fold decrease in MICs for different antibiotics including carbapenem (meropenem) (23). These two studies established the binding of carbapenems to AdeB. One of the most common inhibitors of several RND pumps is Phe-Arg-β-naphthylamide (PAβN). The inhibitory role of PAβN was established by a study that described the crystal structures of the AcrB with PaβN, where PAβN was bound to the wall of the central cavity as well as to the periplasmic site of the pump (24). RND pump inhibition by PAβN was also reinforced by nitrocefin efflux assay where PAβN changed the nitrocefin kinetics into a sigmoidal one, suggesting its inhibitory role. PAβN inhibited the efflux of other drugs by binding to the bottom of the distal binding pocket and also by interfering with the binding of other drug substrates to the upper part of the binding pocket (25).

When an efflux pump encounters a drug, the pump briefly adopts an open conformation accompanied by a contraction to promote the expulsion of the substrate through the chamber and closes immediately after the drug molecule is expelled. The fully assembled efflux pump is observed in a closed state. Although several substrates of AdeB are already known, none of the previous studies had looked into the binding of different carbapenems to AdeB. Structure-based *in-silico* approaches are required to predict how efflux of carbapenems are affected by the chemical structure and physical properties of the molecules (C-2 substituted functional groups) to better understand the contribution of AdeABC efflux pumps in carbapenem resistance among A. baumannii. In this study, the recently published cryo-EM structures of AdeB from A. baumannii were used for the open state and closed state of AdeB to investigate the interaction of different carbapenems and efflux pump inhibitor (EPI) with AdeB (26). We investigated both the interaction of six FDA-approved carbapenems (imipenem, meropenem, ertapenem, doripenem, biapenem, tebipenem) and EPI PAβN with AdeB at three different binding sites (periplasmic site, proximal site, distal site) in both the open and closed state.

The AdeABC efflux pump is regulated by AdeRS, a two-component system (TCS) consisting of a sensor kinase (AdeS) and a response regulator (AdeR) (27). Previously, studies had shown the association of AdeABC efflux pump with carbapenem resistance in A. baumannii (28–30); however, these studies lacked in-depth analysis about the role of AdeRS in carbapenem resistance. Previous publications have shown different mutations within AdeRS associated with tigecycline, ciprofloxacin, or gentamicin resistance in A. baumannii (31–37). In this study, homology modeling was used to investigate the role of the novel mutations within AdeRS in carbapenem resistance. Since, information about the role of AdeRS in carbapenem resistance among A. baumannii is still limited, it is important to know the association of novel mutations in AdeRS with overexpression of AdeABC efflux pump and carbapenem resistance.

Prompted by these considerations, the work presented here was framed to investigate (i) possible atomic level interactions between selected carbapenems and the EPI (PAβN) with AdeB of A. baumannii using molecular docking, and (ii) the mutations within the regulator AdeRS in carbapenem-resistant neonatal septicemic A. baumannii and the structure-related effect of the variants using predictive modeling.

## RESULTS

### Binding of carbapenems and efflux pump inhibitor PAβN with AdeABC efflux pump.

**FIG 1:**
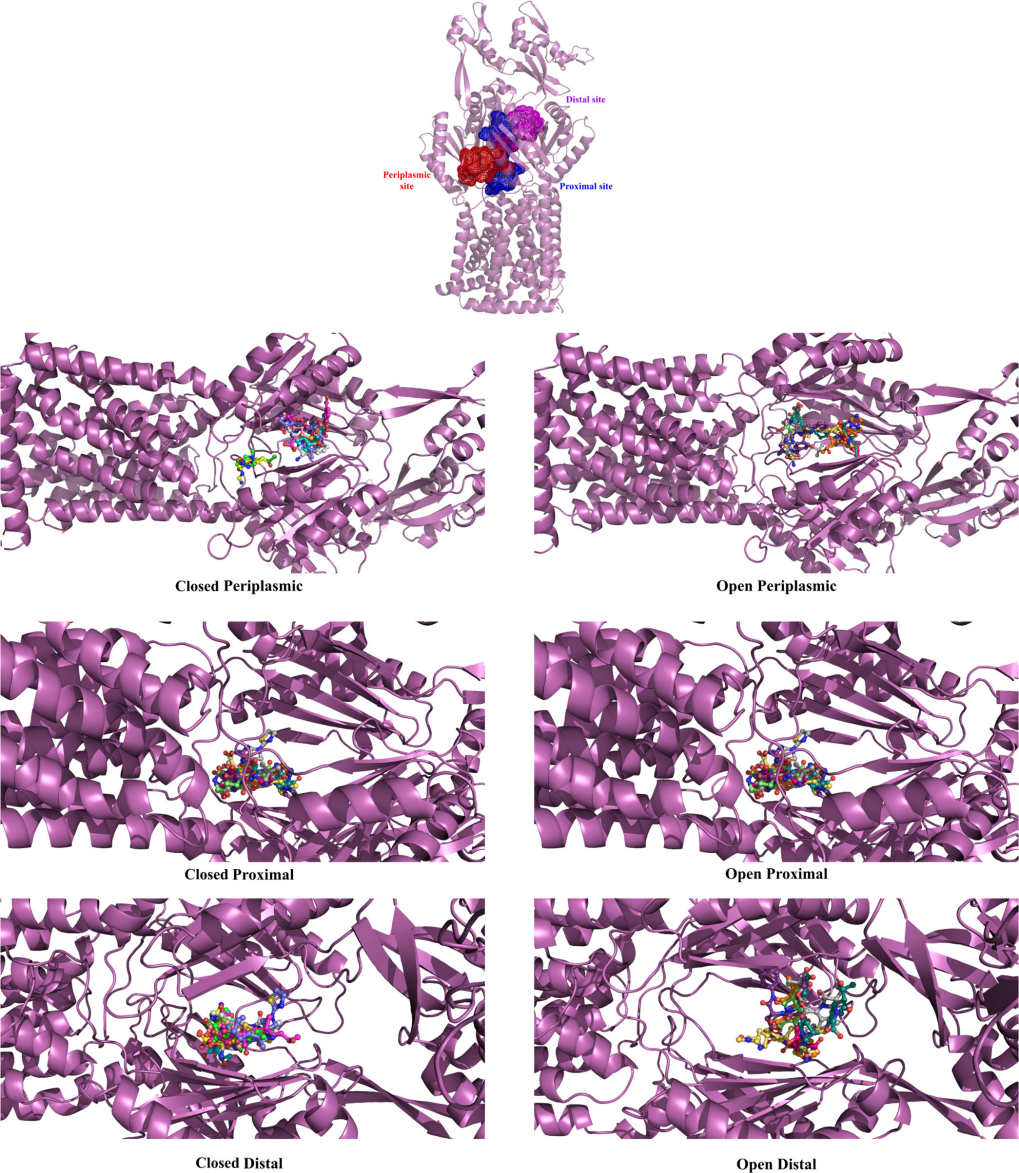
The periplasmic, proximal, and distal sites of AdeB pump. Molecular docking orientations at these sites with substrates (carbapenem or PAβN) for open (right side) and closed (left side) of AdeB. The lowest energy minimized structures were extracted from the result, and the complexes were prepared. The visualization and figure formation were carried out by PyMOL. Substrates are shown in ball-stick representation with all carbons in different colors.

We have explored the possibility of molecular interaction between the amino acids of the AdeB pump and the six FDA-approved carbapenems: imipenem, meropenem, ertapenem, doripenem, biapenem, and tebipenem, along with efflux pump inhibitor PAβN. Half of the selected carbapenems (namely, imipenem, meropenem, ertapenem, doripenem) are currently in clinical use. The docking of EPI PAβN, with the AdeB pump was performed to compare it with the above-mentioned carbapenems. Molecular docking studies were performed targeting three regions (periplasmic, proximal, and distal) of the pump in both open and closed conformations (Fig. 1). Generally, in the multidrug binding site, there are three clefts, first a periplasmic cleft through which the compound (carbapenems or PAβN) enters the efflux pump, and then the proximal site where the compound binds. At the end, the small molecule travels via the G-loop to the distal site of the protein for extrusion (22). The protein–ligand interaction study at the 3 sites had been performed by gathering the grid information (22, 25). The residues used to generate the periplasmic site grid were PHE612, MET656, VAL658, MET706, TRP708, and ILE821. Similarly, for the proximal site, the F-loop residues PRO661-THE668 and residues TRP610, PHE612, SER613, and ALA615 were used for grid preparation. Lastly, the grid for the distal site was prepared from hydrophobic patch residues PHE178, PHE277, ILE279, ILE607, and TRP610. This knowledge-based docking resulted in different conformations, and the structure with minimum energy was selected. The predicted binding energy for each site is shown in Table 1. The binding energy of each carbapenem was found to be better in the open conformation with respect to the closed conformation of AdeB. The periplasmic and proximal sites had shown higher binding energy compared to the distal site in both open and closed conformations. The binding energies of carbapenems in the periplasmic, proximal, and distal regions were −8.6 to −5.5 kcal/mol, −8.0 to −6.2 kcal/mol, and −7.5 to −5.5 kcal/mol respectively for open conformation and −5.6 to −2.0 kcal/mol, −7.6 to −6.3 kcal/mol, and −6.8 to −5.1 kcal/mol for closed conformation. The binding energy of PAβN was higher than the binding energy of carbapenems for both conformations. (Table 1). Since open conformation facilitates the entry of drugs in multidrug resistance systems, the results of these systems are elaborated in detail.

**TABLE 1:** The binding energy of carbapenems and efflux pump inhibitor phenylalanine arginine β-naphthylamide (PAβN) at the periplasmic, proximal, and distal binding sites of AdeABC pump

	Periplasmic site Binding energy (in *kcal/mol*)		Proximal site Binding energy (in *kcal/mol*)		Distal site Binding energy (in *kcal/mol*)	
Conformation	Closed	Open	Closed	Open	Closed	Open
Biapenem	–5.2	–6.0	–6.5	–6.9	–6.0	–6.1
Doripenem	–3.9	–6.8	–7.4	–6.8	–6.7	–6.4
Ertapenem	–3.9	–8.6	–7.6	–8.0	–6.8	–7.5
Imipenem	–5.6	–5.5	–6.3	–5.9	–5.1	–5.5
Meropenem	–4.6	–6.4	–6.8	–7.3	–5.9	–6.3
Tebipenem	–2.0	–6.4	–7.0	–7.5	–6.0	–6.4
PAβN	–6.1	–7.6	–7.6	–9.3	–7.7	–8.9

The open structure clearly shows better binding at the periplasmic site than that of the closed form of AdeB. In the periplasmic region, there was a shift in loops having residues LYS703-GLY715 and ILE821-SER828 that showed deviation in the closed and open conformation (Fig. S1 in the supplemental material). In open conformation, these shifts allow carbapenems to enter the efflux pump. In the open structure, the AdeB and carbapenem interaction showed that residues GLU563, SER572, PHE573, GLN574, PHE612, SER613, MET656, ALA657, LEU659, THE668, GLY671, SER673, TRP708, ASN709, GLU710, GLY711, and GLY820 at the periplasmic site interact with at least one carbapenem. However, among the carbapenems, meropenem and tebipenem showed comparatively less interactions with AdeB residues. The β-lactam ring of the carbapenem was found to interact with GLY820 for doripenem; the main chain of GLN574 interacts with meropenem and imipenem; imipenem also may interact with PHE573; and GLU710 interacts with tebipenem. (Table S1). The inhibitor PAβN interacted with GLU563, LEU659, PRO661, and THR668. The aromatic rings in PAβN were found to cover active site residues such as PHE612, TRP708, ILE821, and others (Fig. S2, Table S2). This infers that the carbapenems were interacting with the periplasmic region of AdeB, leading to carbapenem resistance through this efflux pump. The inhibitor PAβN also has shown a tendency to bind to the entrance site of the efflux pump.

10.1128/msystems.00217-22.1 FIG S1 The interaction of six FDA-approved carbapenems (imipenem, meropenem, ertapenem, doripenem, biapenem, and tebipenem) with the periplasmic site in open (right column) and closed (left column) state of AdeABC efflux pump. The protein is displayed in cartoon representation. The carbapenems are represented in ball-stick form, and interacting residues are represented in stick form. Download FIG S1, TIF file, 2.6 MB. Copyright © 2022 Roy et al. 2022 Roy et al. https://creativecommons.org/licenses/by/4.0/ This content is distributed under the terms of the Creative Commons Attribution 4.0 International license.

10.1128/msystems.00217-22.2 FIG S2 The interaction of the efflux pump inhibitor phenylalanine arginine β-naphthylamide (PAβN) with the periplasmic, proximal and distal sites in open (right column) and closed (left column) state of the AdeABC efflux pump. The protein is displayed in cartoon representation. PAβN is represented in ball-stick form, and interacting residues are represented in stick form. Download FIG S2, TIF file, 2.3 MB. Copyright © 2022 Roy et al. 2022 Roy et al. https://creativecommons.org/licenses/by/4.0/ This content is distributed under the terms of the Creative Commons Attribution 4.0 International license.

10.1128/msystems.00217-22.8 TABLE S1 Details of molecular interactions between the three different sites (periplasmic, proximal, and distal) of the AdeB protein (open and closed conformation) and six FDA-approved carbapenems (biapenem, doripenem, ertapenem, imipenem, meropenem, and tebipenem). For each carbapenem, the binding site name followed by the carbapenem drug name has been used. The table highlights the distance between interacting atoms (i.e., an atom from residues of AdeB protein and an atom from the carbapenem antibiotic). Download Table S1, DOCX file, 0.04 MB. Copyright © 2022 Roy et al. 2022 Roy et al. https://creativecommons.org/licenses/by/4.0/ This content is distributed under the terms of the Creative Commons Attribution 4.0 International license.

10.1128/msystems.00217-22.9 TABLE S2 Details of molecular interactions between the different sites (periplasmic, proximal, and distal) of the AdeB protein (open and closed conformation) and its inhibitor phenylalanine arginine β-naphthylamide (PAβN). The table highlights the distance between interacting atoms (i.e., an atom from residues of AdeB protein and an atom from the inhibitor PAβN). Download Table S2, DOCX file, 0.03 MB. Copyright © 2022 Roy et al. 2022 Roy et al. https://creativecommons.org/licenses/by/4.0/ This content is distributed under the terms of the Creative Commons Attribution 4.0 International license.

At the proximal site, residues ASN44, ASP83, THE87, GLU89, GLN128, ALA180, GLU181, GLN273, TRP610, GLY611, PHE612, SER613, GLY614, and ALA615 interact with at least one carbapenem (Fig. S3, Table S1). These residues generate a cleft like structure in which most of the residues were electronegative and the residues at the end of the cleft were electropositive in nature. The β-lactam rings of tebipenem and meropenem were found to interact with THR329 and PHE35 residues, respectively, at the proximal cleft. The inhibitor PAβN was observed to have possible interactions with residues TRP610, GLY611, PHE612, SER613, and GLY614, which are G-loop residues (Fig. S2, Table S2). This indicates that it has stronger binding to this site and hence blocks the cleft for carbapenem.

10.1128/msystems.00217-22.3 FIG S3 The interaction of six FDA-approved carbapenems (imipenem, meropenem, ertapenem, doripenem, biapenem, and tebipenem) with the proximal site in open (right column) and closed (left column) state of the AdeABC efflux pump. The protein is displayed in cartoon representation. The carbapenems are represented in ball-stick form, and interacting residues are represented in stick form. Download FIG S3, TIF file, 2.6 MB. Copyright © 2022 Roy et al. 2022 Roy et al. https://creativecommons.org/licenses/by/4.0/ This content is distributed under the terms of the Creative Commons Attribution 4.0 International license.

For the distal cleft, the residues interacting with at least one carbapenem were ARG34, PHE35, SER37, SER133, SER134, PHE136, GLN176, TYR327, THE329, GLN566, PRO661, ALA662, ILE663, ASP664, and GLU665 (Fig. S4, Table S1). The β-lactam ring-interacting residues found in the distal cleft were ILE663 for biapenem, ALA662 for biapenem and ertapenem, PRO661 for doripenem and meropenem, ASP664 for ertapenem, and SER133 for tebipenem (Table S1). In the distal cleft, the PAβN interacting residues were ARG34, SER134, TYR327, GLN566, PRO661, ALA662, ILE663, ASP664, and GLU665 (Fig. S2, Table S2).

10.1128/msystems.00217-22.4 FIG S4 The interaction of six FDA-approved carbapenems (imipenem, meropenem, ertapenem, doripenem, biapenem, and tebipenem) with the distal site in open (right column) and closed (left column) state of the AdeABC efflux pump. The protein is displayed in cartoon representation. The carbapenems are represented in ball-stick form, and interacting residues are represented in stick form. Download FIG S4, TIF file, 2.6 MB. Copyright © 2022 Roy et al. 2022 Roy et al. https://creativecommons.org/licenses/by/4.0/ This content is distributed under the terms of the Creative Commons Attribution 4.0 International license.

### Bacterial isolates and carbapenem resistance.

A total of 55 A. baumannii were isolated from the blood of septicemic neonates, of which 69% (38/55) were resistant to meropenem and imipenem (Table 2). The MIC_90_ of meropenem and imipenem was 64 mg/L and 128 mg/L, respectively. Carbapenemases (oxacillinases and metallo-β-lactamases) were detected among the carbapenem-resistant A. baumannii (*n* = 38). The prevalence of OXA-23-like, OXA-58-like, and NDM-1 was 95% (*n* = 36), 11% (*n* = 4), and 8% (*n* = 3), respectively (Table 2). This part of the results has already been published (15).

**TABLE 2:** MIC for carbapenems (meropenem and imipenem) with/without efflux pump inhibitor phenylalanine arginine β-naphthylamide (PAβN) in Acinetobacter baumannii (*n* = 55) isolated during 2007–2015 along with the presence of carbapenemases in the carbapenem-resistant A. baumannii (*n* = 38)^*a*^

Strain no.	Meropenem MIC (mg/L)	Meropenem + PAβN (mg/L)	Meropenem fold change	Imipenem MIC (mg/L)	Imipenem + PAβN (mg/L)	Imipenem fold change	Presence of carbapenemases
A_101	0.5	0.25	2-fold	1	0.38	2.6-fold	ND
A_102	2	1	2-fold	2	1	2-fold	ND
A_103	0.25	0.125	2-fold	0.25	0.125	2-fold	ND
A_104	1	1	No fold change	2	1	2-fold	ND
A_105	16	4	**4-fold**	16	8	2-fold	OXA-51-like, OXA-23-like
A_106	1	0.5	2-fold	1	0.5	2-fold	ND
A_107	16	8	2-fold	16	8	2-fold	OXA-51-like, OXA-23-like
A_108	16	4	**4-fold**	16	8	2-fold	OXA-51-like, OXA-23-like
A_112	0.25	0.125	2-fold	0.25	0.125	2-fold	ND
A_113	1	0.5	2-fold	1	0.5	2-fold	ND
A_115	32	32	No fold change	64	32	2-fold	OXA-58-like, NDM-1
A_117	0.5	0.5	No fold change	0.25	0.125	2-fold	ND
A_118	4	2	2-fold	4	2	2-fold	ND
A_120	0.5	0.25	2-fold	1	0.5	2-fold	ND
A_123	32	16	2-fold	32	32	No fold change	OXA-51-like, OXA-23-like
A_124	0.5	0.25	2-fold	0.25	0.25	No fold change	ND
A_125	2	1	2-fold	2	1	2-fold	ND
A_126	0.5	0.25	2-fold	0.5	0.25	2-fold	ND
A_130	16	4	**4-fold**	32	8	**4-fold**	OXA-51-like, OXA-23-like
A_131	8	8	No fold change	32	16	2-fold	OXA-51-like, OXA-23-like
A_132	0.5	0.25	2-fold	0.5	0.25	2-fold	ND
A_133	16	2	**8-fold**	32	4	**8-fold**	OXA-51-like, OXA-23-like
A_134	32	16	2-fold	32	16	2-fold	OXA-51-like, OXA-23-like, OXA-58-like
A_135	8	2	**4-fold**	16	4	**4-fold**	No carbapenemases present
A_136	8	0.25	**32-fold**	8	2	**4-fold**	OXA-51-like, OXA-23-like, OXA-58-like
A_138	2	0.75	2.6-fold	2	0.75	2.6-fold	ND
A_141	64	32	2-fold	64	32	2-fold	OXA-51-like, OXA-23-like
A_145	64	32	2-fold	256	16	**16-fold**	OXA-51-like, OXA-23-like
A_146	1	1	No fold change	1	1	No fold change	ND
A_147	64	32	2-fold	32	32	No fold change	OXA-51-like, OXA-23-like
A_149	32	16	2-fold	32	8	**4-fold**	ND
A_150	16	8	2-fold	16	8	2-fold	OXA-51-like, OXA-23-like
A_151	32	16	2-fold	32	16	2-fold	OXA-51-like, OXA-23-like
A_152	32	16	2-fold	32	16	2-fold	OXA-51-like, OXA-23-like
A_153	32	16	2-fold	32	16	2-fold	OXA-51-like, OXA-23-like
A_155	64	16	**4-fold**	64	16	**4-fold**	OXA-51-like, OXA-23-like
A_158	32	16	2-fold	32	16	2-fold	OXA-51-like, OXA-23-like
A_159	32	16	2-fold	32	16	2-fold	OXA-51-like, OXA-23-like
A_160	32	16	2-fold	32	16	2-fold	OXA-51-like, OXA-23-like
A_161	64	64	No fold change	64	32	2-fold	OXA-51-like, OXA-23-like
A_162	16	8	2-fold	64	16	**4-fold**	OXA-51-like, OXA-23-like
A_163	32	8	**4-fold**	16	8	2-fold	OXA-51-like, OXA-23-like
A_166	64	64	No fold change	128	64	2-fold	OXA-51-like, OXA-23-like
A_167	64	8	**4-fold**	64	8	**8-fold**	OXA-51-like, OXA-23-like, OXA-58-like
A_168	32	8	**4-fold**	32	32	No fold change	OXA-51-like, OXA-23-like, NDM-1
A_169	64	16	**4-fold**	16	8	2-fold	OXA-51-like, OXA-23-like
A_170	128	64	2-fold	64	16	**4-fold**	OXA-51-like, OXA-23-like
A_171	32	16	2-fold	16	8	2-fold	OXA-51-like, OXA-23-like
A_172	64	32	2-fold	32	16	2-fold	OXA-51-like, OXA-23-like
A_173	64	32	2-fold	32	16	2-fold	OXA-51-like, OXA-23-like
A_176	64	32	2-fold	32	16	2-fold	OXA-51-like, OXA-23-like
A_177	0.5	0.5	No fold change	0.125	0.125	No fold change	ND
A_178	256	256	No fold change	128	128	No fold change	OXA-51-like, OXA-23-like
A_179	32	16	2-fold	4	4	No fold change	OXA-51-like, OXA-23-like
A_180	32	16	2-fold	4	4	No fold change	OXA-51-like, OXA-23-like, NDM-1

a ≥4-fold reduction in MIC of carbapenems (meropenem and/or imipenem) in the presence of PAβN, is indicated as bold. ND = carbapenemases were not detected as these strains were carbapenem susceptible.

### Assessment of expression of AdeABC efflux pumps among CRABs.

Exposure of the CRABs (*n* = 38) with PAβN resulted in a drastic reduction (4- to 32-fold MIC decreases) in the MICs of carbapenems among 39% of isolates (15/38), indicating the role of multidrug efflux pumps in carbapenem resistance (Table 2). On the other hand, no significant increase in susceptibility was found for carbapenem susceptible isolates (Table 2). mRNA expression of *adeB* was analyzed by quantitative reverse transcriptase PCR (qRT-PCR) for the isolates (*n* = 15), which showed ≥4-fold reduction of MIC for meropenem and/or imipenem in the presence of PAβN. A total of 11 out of 15 CRABs showed overexpression of *adeB* (2.2 to 42.8-fold) in comparison to the control A. baumannii ATCC19606 (Table 3). Overall, 29% (11/38) of total CRAB showed overexpression of AdeABC efflux pump.

**TABLE 3:** Carbapenem MIC with/without efflux pump inhibitor phenylalanine arginine β-naphthylamide (PAβN), expression level (fold change) of *adeB* gene, and mutations in the pumps’ regulator AdeRS among A. baumannii isolates (*n* = 15), which showed ≥4-fold reduction in MIC of carbapenems (meropenem and/or imipenem) in the presence of PAβN^*a*^

Strain no.	STs	Meropenem MIC (mg/L)	Meropenem + PAβN (mg/L)	Meropenem fold change	Imipenem MIC (mg/L)	Imipenem + PAβN (mg/L)	Imipenem fold change	*adeB* fold change detected by qRT-PCR	Amino acid changes within AdeRS		
									Changes not associated with overexpression of AdeABC^*b*^		Changes associated with overexpression of AdeABC
									AdeR	AdeS	AdeS
A_105	116	16	4	**4-fold**	16	8	2-fold	**16**	No mutation	ALA94VAL LEU172PRO PHE214LEU TYR303PHE	GLU121LYS
A_108	623	16	4	**4-fold**	16	8	2-fold	**6.6**	No mutation	PHE214LEU LEU172PRO TYR303PHE	GLU121LYS
A_130	575	16	4	**4-fold**	32	8	**4-fold**	0.06	ND	ND	ND
A_133	1406	16	2	**8-fold**	32	4	**8-fold**	0.04	ND	ND	ND
A_135	undetermined	8	2	**4-fold**	16	4	**4-fold**	1.4	ND	ND	ND
A_136	149	8	0.25	**32-fold**	8	2	**4-fold**	**5**	VAL120ILE	LEU172PRO TYR303PHE ILE331VAL	SER188PHE VAL255ILE
A_145	149	64	32	2-fold	256	16	**16-fold**	**12.3**	No mutations	LEU172PRO TYR303PHE LEU322PHE ILE331VAL SER341CYS	SER188PHE VAL255ILE
A_149	149	32	16	2-fold	32	8	**4-fold**	**2.2**	VAL120ILE	LEU172PRO TYR303PHE ILE331VAL SER341CYS	SER188PHE, VAL255ILE
A_155	10	64	16	**4-fold**	64	16	**4-fold**	**5.4**	No mutations	LEU172PRO PHE214LEU TYR303PHE LEU322PHE	GLU121LYS
A_162	1406	16	8	2-fold	64	16	**4-fold**	**17.5**	No mutations	LEU172PRO TYR303PHE	GLY186VAL
A_163	575	32	8	**4-fold**	16	8	2-fold	**20**	VAL120ILE ALA136VAL	LEU172PRO TYR303PHE	GLY186VAL
A_167	2	64	8	**4-fold**	64	8	**8-fold**	**25.3**	VAL120ILE ALA136VAL	LEU172PRO TYR303PHE	GLY186VAL
A_168	149	32	8	**4-fold**	32	32	No fold change	**2.3**	VAL120ILE	LEU172PRO TYR303PHE ILE331VAL	SER188PHE, VAL255ILE
A_169	976	64	16	**4-fold**	16	8	2-fold	0.05	ND	ND	ND
A_170	2	128	64	2-fold	64	16	**4-fold**	**4.8**	VAL120ILE ALA136VAL	TYR303PHE ILE331VAL	GLY186VAL

a ≥4-fold reduction in MIC of carbapenems (meropenem and/or imipenem) in the presence of PAβN, is indicated as bold. ST, sequence type ND, sequencing of AdeRS was not done as the strains did not show overexpression of AdeB,

b These mutations were also detected in the reference strains (A. baumannii ACICU, A. baumannii AYE, ATCC 17978, and ATCC 19606), which were used for sequence comparison. Thus, these amino acid changes seem not to be associated with overexpression of AdeABC.

### Structural analysis of AdeRS and role of novel mutations in carbapenem resistance.

**FIG 2:**
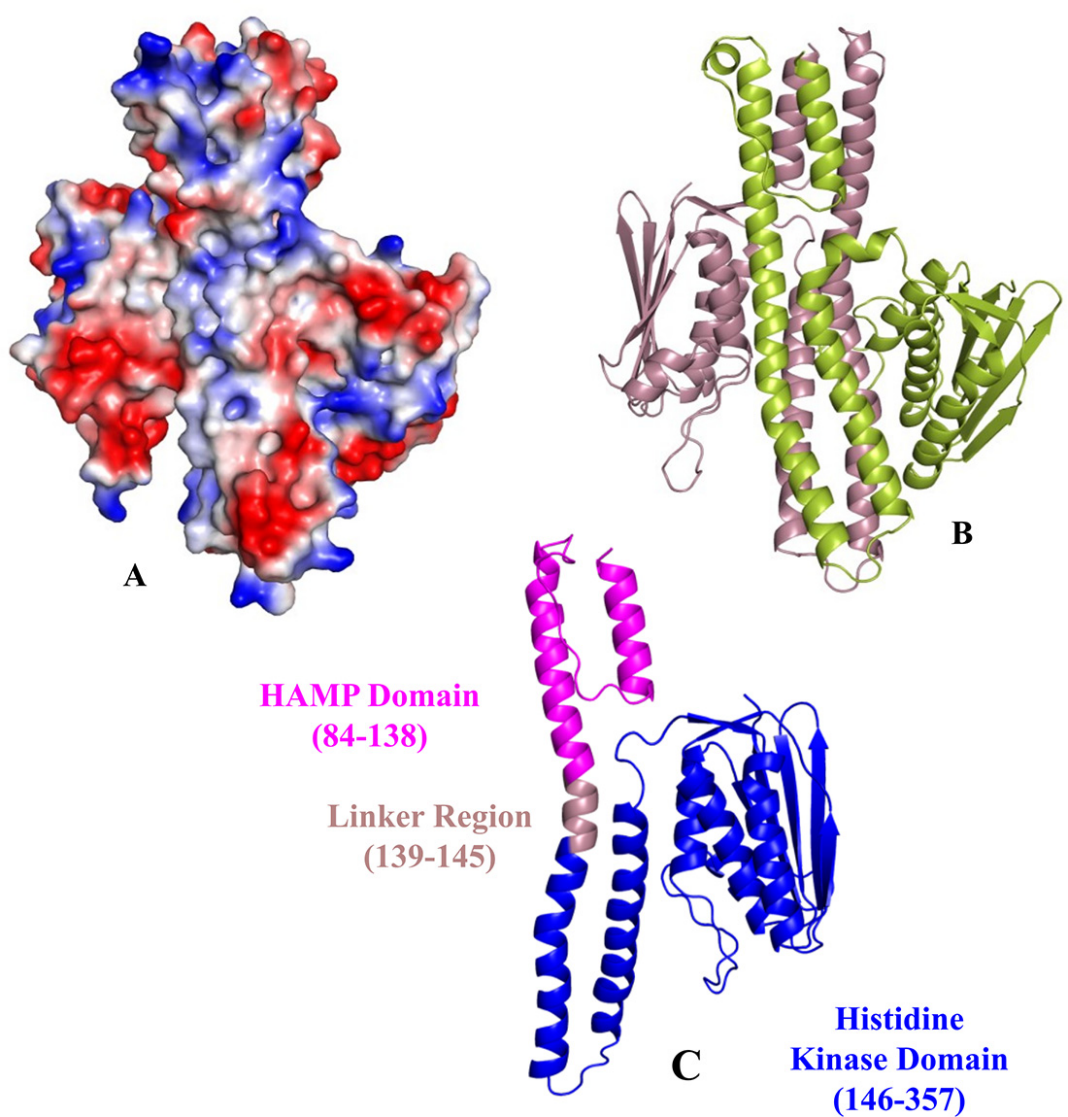
Homology modeling of the cytosolic region of AdeS. (A) Representation of the electrostatic surface orientation: red color indicates negatively charged residues; blue color indicates positively charged residues; and the rest in white are neutral residues. (B) Cartoon representation of AdeS homodimer where monomers are represented in different colors. (C) Monomeric structure of the cytosolic region of AdeS. The domains of this region have been highlighted with different colors: HAMP domain (histidine kinases, adenyl cyclases, methyl-accepting proteins, phosphatases) in magenta, HK domain (histidine kinase) in blue, and the rest denotes linking region.

**FIG 3:**
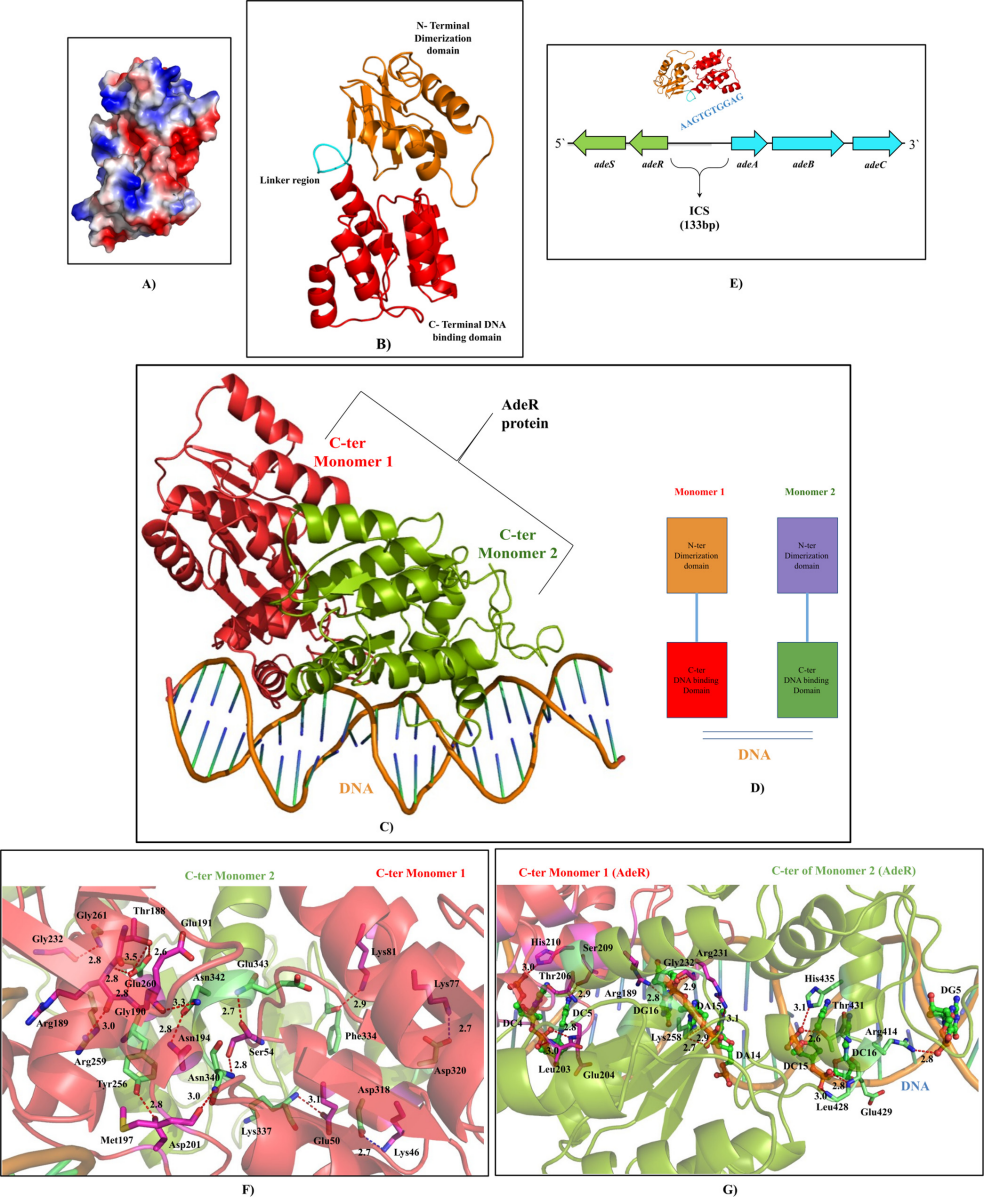
Homology modeling of the AdeR in monomer form. (A) Representation of the electrostatic surface orientation: red color indicates negatively charged residues, blue color represents positively charged residues, and rest in white are neutral residues. (B) Cartoon representation of AdeR domains: the C-terminal (DNA binding domain) in red and N-terminal (dimerization domain/receiver domain) in orange. (C) AdeR binds to the intercistronic spacer (ICS) region. (D and E) Binding of C-terminus of monomer 1 (red) and monomer 2 (green) of AdeR dimer with DNA. (F) C-terminus domain of the AdeR binds to DNA after being phosphorylated. The interactions between DNA and the two C-terminal domains of AdeR is described here. (G) Interaction between AdeR and DNA in the DNA binding domain.

Overexpression of the efflux pump results in the transport of carbapenems to the outside of the bacterial cell (carbapenem binding site has been observed in the structural study of the pump). AdeRS (TCS) acts as the regulator of the AdeABC pump and is associated with the altered expression of the pump. Hence, mutations (detected by sequencing) within AdeRS leading to carbapenem resistance have been investigated. AdeS is a sensor histidine kinase (Fig. 2), and AdeR a response regulator (Fig. 3). AdeS and AdeR are located upstream of AdeA and transcribed in the opposite direction (Fig. 3). When AdeS receives an environmental signal, it induces auto-phosphorylation through its histidine kinase domain and the phosphate group is then transferred to the response regulator. The phosphoryl signal to AdeR results in the expression of its target genes (38).

### Structural analysis.

A homology model was built for the AdeS, that has around 357 amino acid residues comprising the transmembrane, HAMP (histidine kinases, adenyl cyclases, methyl-accepting proteins, phosphatases), and HK domain (histidine kinase). The HK domain has been found to be involved in the phosphorylation process. The structure of this HK is in a homodimeric form. The predicted structure using E. coli HK as a template clearly shows the dimeric nature. The cytoplasmic region of AdeS HK has been divided into two major regions: N-terminal DHp domain (dimerization and histidine-containing phosphotransfer domain) and C-terminal CA domain (catalytic and ATP-binding domain). The DHp domain, which contains the autophosphorylation site, forms a stable dimer and can be phosphorylated in the presence of ATP by the CA domain. The active form of AdeS, i.e., an auto-phosphorylated state, has the asymmetric conformation of the homodimer (Fig. 2).

As in case of AdeS, a homology model was also built for AdeR. AdeR has two domains linked by a connecting loop. The N-terminal and C-terminal domains are the dimerization/receiver and DNA binding domains (DBD), respectively. The N-terminal receiver domain of AdeR forms a dimer using the 4–5–5 motif ASP108, PHE109, and ARG122, developing a toothed wheel type structure by stack cation interaction. It has been reported that mutation of these critical amino acid residues not only breaks the dimerization motif of AdeR but also reduces its interaction with the intercistronic spacer (ICS) region. According to current literature, AdeR recognizes an ICS region (133 bp) and binds to a 10 bp direct repeat, AAGTGTGGAG, within the central 30 bp of the ICS region which is located between AdeR and AdeA. This region lies between the AdeR and efflux pump AdeABC operon (Fig. 3) (38). The full-length AdeR could recognize and develop an interaction with the ICS region with an affinity (Kd of 20 nM), 100 times higher than the separated DNA binding domain of AdeR. More interestingly, the conserved DNA binding domain of the AdeR protein (OmpR/PhoB-like) uses the long 3 helix and the beta-hairpin formed by β-5 and β-6 to bind both the major as well as minor groove of the ICS region, respectively. In this study, the interaction between two C-terminal monomers (monomer1 and monomer2) of AdeR and DNA was analyzed. It was observed that the residues LYS46, GLU50, SER54, LYS77, LYS81, THR188, ARG189, GLY190, GLU191, ASN194, MET197, ASP201, and GLY232 of monomer1 had made around 15 hydrogen bonds with monomer2 of AdeR (Table 4). Additionally, the residues ARG189, ARG231, GLY232, LEU203, GLU204, THR206, SER209, and HIS210 of monomer1 interact with DNA. Of these 8 residues, ARG189, GLY232, LEU203, GLU204, HIS210 interact with the backbone of the DNA helix (Table 5). Similarly, in the case of monomer 2 of AdeR, residues LYS258, ARG414, LEU428, GLU429, THR431, and HIS435 interact with only the backbone of DNA (Fig. 3).

**TABLE 4:** Details of molecular interactions between the two monomers of AdeR protein

Monomer1		Monomer2		Distance (Å)
Residue	Atom	Residue	Atom	
LYS46	NZ	ASP318	OD2	2.70
GLU50	OE2	LYS337	N	3.06
SER54	O	GLU343	N	2.70
	OG	ASN340	N	2.76
LYS77	NZ	ASP320	OD2	2.68
LYS81	NZ	PHE334	O	2.94
THR188	OG1	GLU260	OE1	2.65
ARG189	N	GLU260	OE2	2.79
	O	ARG259	NH2	2.96
GLY190	N	GLU260	OE2	2.80
GLU191	O	ASN342	ND2	3.29
ASN194	ND2	ASN342	OD1	2.85
MET197	O	ASN340	ND2	2.98
ASP201	OD2	TYR256	OH	2.77
GLY232	O	GLY261	N	2.82

**TABLE 5:** Details of molecular interactions between the dimer of AdeR protein and DNA

AdeR protein		DNA		Distance (Å)
Monomer1		5′ to 3′		
Residue	Atom	Residue	Atom	
ARG189	NH1	DG16	O2P	2.78
ARG231	NH2	DA14	N3	3.07
GLY232	N	DG16	O1P	2.88
		**3′ to 5′**		
LEU203	N	DC5	O1P	2.99
GLU204	N		O2P	2.78
THR206	OG1	DC4	O2P	3.08
THR206	OG1	DC4	O5’	3.21
SER209	OG	DC5	N4	2.90
HIS210	NE2	DC4	O2P	3.00
**Monomer2**		**5′ to 3′**		
LYS258	NZ	DA15	O2P	2.75
LYS258	NZ	DA15	O5’	2.90
ARG414	NH1	DG5	O2P	2.84
		**3′ to 5′**		
LEU428	N	DC16	O1P	2.98
GLU429	N	DC16	O2P	2.78
THR431	OG1	DC15	O2P	2.63
HIS435	NE2	DC15	O2P	3.08

### Novel mutations.

In this study, four different mutations were observed in AdeS, among which three were in the dimerization domain of AdeS (GLY186VAL [*n* = 4], SER188PHE [*n* = 4], GLU121LYS [*n* = 3]) and one in the catalytic domain of AdeS (VAL255ILE [*n* = 4]) (Table 3). These mutations were not detected in the reference strains (A. baumannii ATCC 19606, A. baumannii ATCC 17978, A. baumannii AYE, A. baumannii ACICU). It has been reported that the mutation in AdeS leads to overexpression of AdeB efflux pump, resulting in drug resistance (38). Hence, to correlate the possible effects of these mutations we have performed structure-based analysis of the mutations in AdeS.

**FIG 4:**
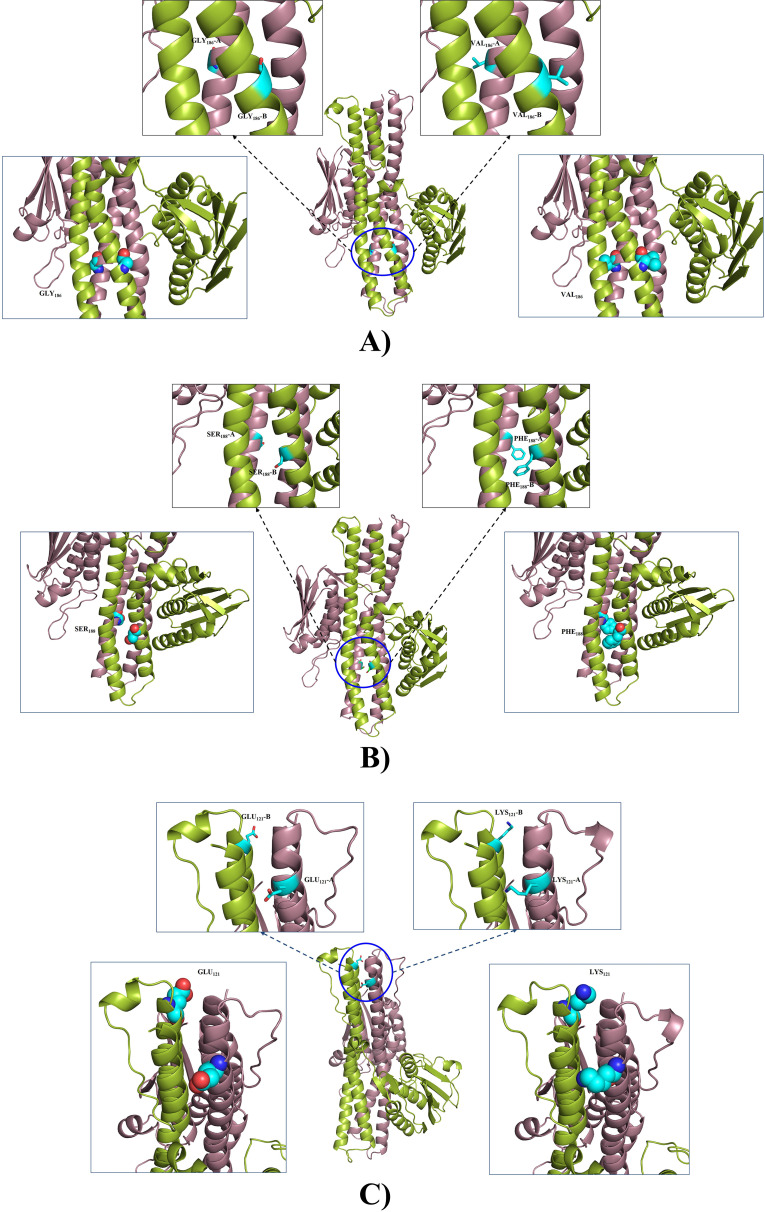
The position of GLY_186_VAL, SER_188_PHE, and GLU_121_LYS mutations in AdeS homodimer. The mutational residues are represented in cyan. (A) GLY_186_ in wild-type AdeS residue (upper left box), and its mutant VAL_186_ (upper right box); the lower boxes represent the spatial orientation of wild-type residue GLY_186_ (lower left box) and its mutant VAL_186_ (lower right box) as spheres. (B) SER_188_ in wild-type AdeS residue (upper left box), and its mutant PHE_188_ (upper right box). The lower boxes represent the spatial orientation of wild-type residue SER_188_ (lower left box) and its mutant PHE_188_ (lower right box) as spheres. (C) GLU_121_ in wild-type AdeS residue (upper left box), and its mutant LYS_121_ (upper right box). The lower boxes represent the spatial orientation of wild type residue GLU_121_ (lower left box) and its mutant LYS_121_ (lower right box) as spheres.

The mutation of GLY186 is located in the dimerization domain, and the alpha-helix containing this residue is near the DHp domain of AdeS. The DHp domain is in the proximity of histidine residue (HIS149), which is phosphorylated. However, in the study, this glycine residue was mutated to valine. The VAL186 was observed to have a much bigger side chain compared to the glycine residue. Thus, glycine to valine conversion might have imposed van der Waals repulsion in the side chains in the dimerization domain. This could result in the destabilization of the AdeS homodimer, resulting in prevention of auto-phosphorylation in AdeS. This could inhibit/slow the transfer of the phosphate group to AdeR. Thus, AdeR might be binding loosely to the ICS region. It has been reported that the AdeR loose binding in the ICS region leads to an overexpression of AdeABC (38). This could be a probable scenario for this mutation. (Fig. 4A).

The residue SER188 is also found in the dimerization domain of AdeS. It is located in the α-helices which is near the DHp domain. This helical part of the DHp domain is near the phosphorylated histidine residue (HIS149) involved in auto-phosphorylation. The SER188 was found to be mutated to PHE188. The serine residue is hydrophilic in nature. However, phenylalanine is hydrophobic in nature, and it has a much longer side chain with a bulky benzene ring at the end. In the resistant strains, phenylalanine was found in place of serine. Thus, replacement of hydrophilic interaction into hydrophobic interactions, steric hindrance, and van der Waals repulsions due to the bulky side chain probably hinders the dimerization of AdeS homodimer. Therefore, we speculate that this change affects the DHp domain, leading to nonformation of the homodimer of AdeS HK. Hence, in this case, the AdeR binding toward the ICS region could not be hampered, due to which overexpression of AdeABC might have occurred (Fig. 4B).

Another residue, GLU121, also in the dimerization domain of the AdeS homodimer, is in the helix extended from auto-phosphorylating histidine, i.e., HIS149. The GLU121 is situated at the end of the helix where two helical dimer domains of AdeS are present. The mutation observed at this site was LYS121. The GLU121 was found to interact with residues LEU122 and ASN125 from the same monomer (or chain) and LYS98 from another homomer (chain). However, these interactions were absent in the mutated LYS121 residue. The residue GLU121 was found at 2.8 distance from ALA94, and in the mutated version, the distance was extended to 3.5. Thus, the loss of interaction could be observed due to this mutation resulting in a loose end in the dimerization domain and less probability to stabilize the dimerization process of AdeS. An unstable dimerization domain could hamper auto-phosphorylation of AdeS, leading to problems in phospho-transfer to AdeR. Hence, interaction between the AdeR and ICS region occur inadequately, leading to AdeABC overexpression (Fig. 4C).

**FIG 5:**
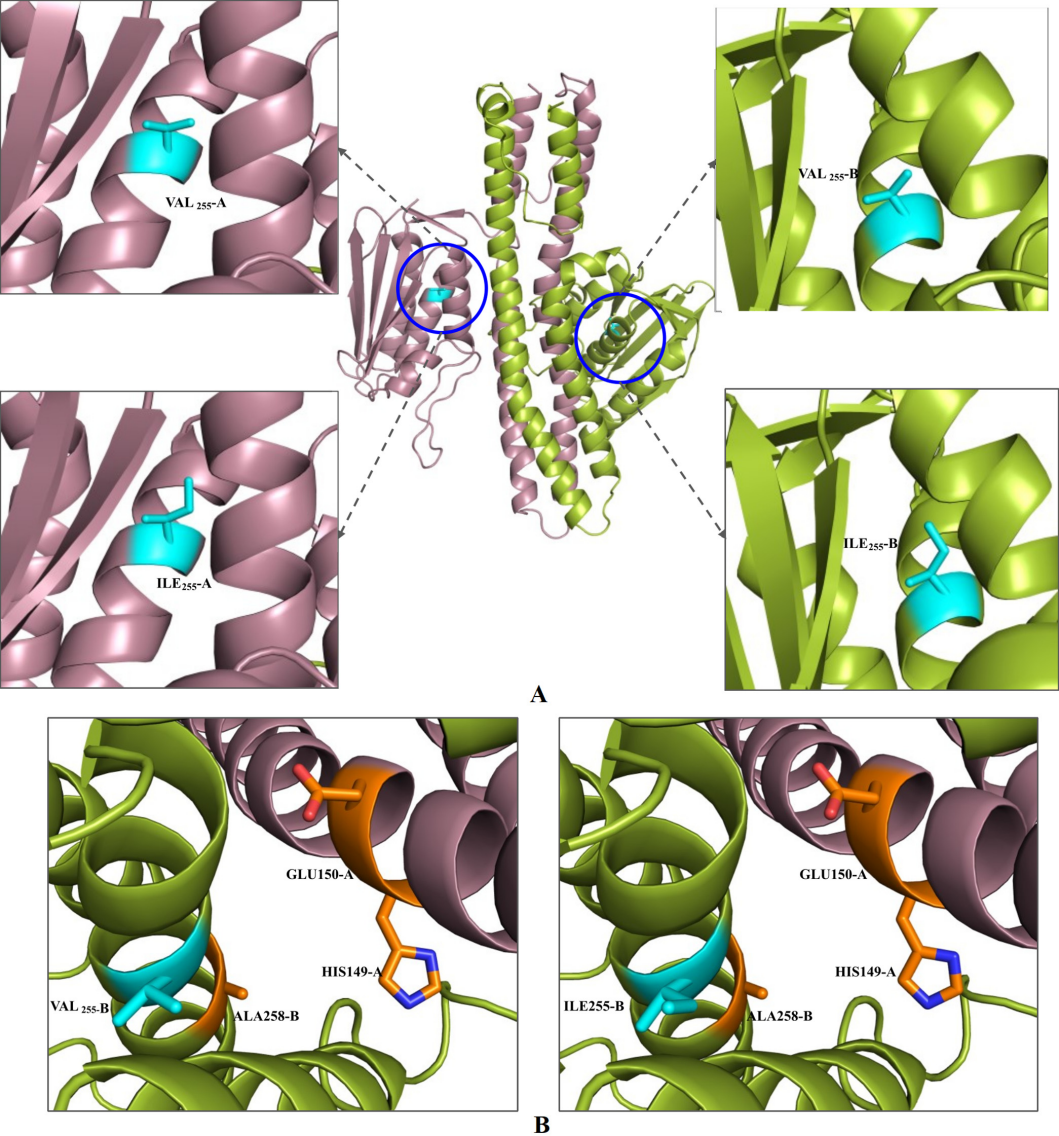
The position of VAL_255_ILE mutation in AdeS homodimer. (A) The mutational residues are represented in cyan: VAL_255_ in wild-type AdeS residue (upper boxes) and its mutant ILE_255_ (lower boxes). (B) The spatial orientation of residue VAL_255_ and its mutant ILE_255_. Both of these residues are represented in cyan, and the Michaelis complex is represented in orange. Position of VAL_255_ with respect to Michaelis complex involved in auto-phosphorylation (left box) and position of ILE_255_ with respect to Michaelis complex involved in auto-phosphorylation (right box).

The VAL255 is located in the catalytic domain of the AdeS, where ATP binding occurs. This residue was found to be near the catalytic residues (HIS149, GLU150, and ALA356) that make the Michaelis complex. The VAL255 is situated at the helix, which also contains catalytic residue ALA258. However, VAL is hydrophobic in nature, and hence it is observed to be buried in the inner side of enzymes. The mutation of VAL to ILE does not have much effect, as ILE is also hydrophobic in nature and occupies the space in a similar volume as VAL. Thus, this mutation probably had not resulted in any conformational effect, not affecting the activity of AdeS (Fig. 5).

**FIG 6:**
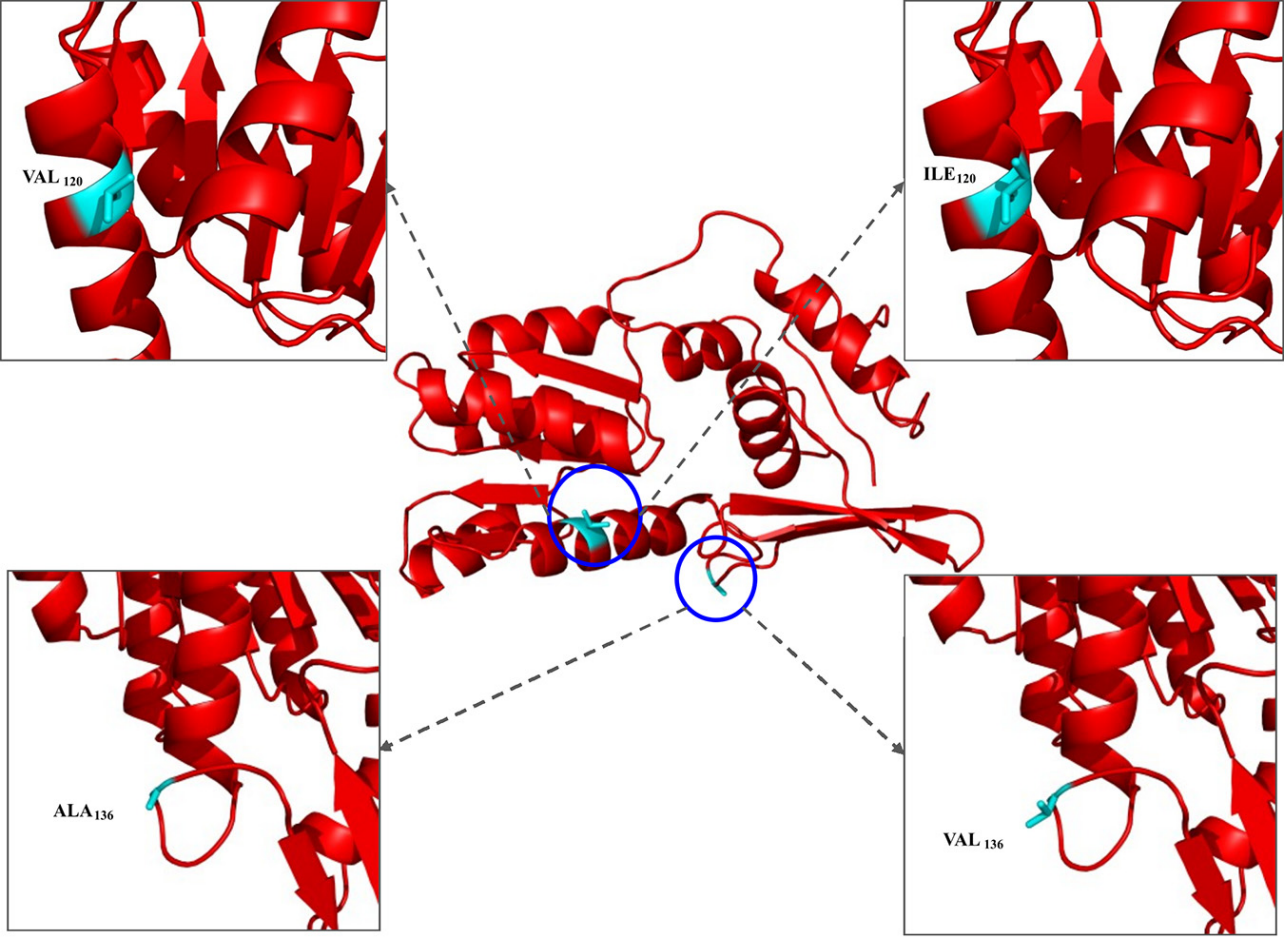
The mutations in the AdeR monomer unit. The residues are shown in yellow. The mutation in the N-terminal domain is VAL_120_ILE, and it is represented in the upper boxes where left box represent the wild-type residue VAL_120_ and the right box represent the mutated residue ILE_120_. The connecting loop mutation is ALA_136_VAL, and it is represented in the lower boxes where the left box represents the wild-type residue ALA_136_ and the right box represent the mutated residue VAL_136_.

In the case of AdeR, two mutations, VAL120ILE (*n* = 7) and ALA136VAL (*n* = 3), were observed in the N-terminal domain and in the connecting loop region, respectively. VAL120ILE mutation was found in the last helix. Valine and isoleucine are both hydrophobic amino acids. Thus, this mutation does not bring about significant changes in the structure. Similarly, in the case of the ALA136VAL mutation, no effect was found, and the reason could be the similar hydrophobic residue mutation in the connecting loop (Fig. 6). Moreover, these two mutations were also detected in the reference strains.

### Mutations within AdeRS in different STs.

Isolates (*n* = 11) that showed overexpression of AdeB belonged to different STs including ST2 (*n* = 2), ST10 (*n* = 1), ST116 (*n* = 1), ST149 (*n* = 4), ST575 (*n* = 1), ST623 (*n* = 1), and ST1406 (*n* = 1) (Table 3). Four novel mutations (GLY186VAL, SER188PHE, GLU121LYS, and VAL255ILE) were detected in AdeS. Of these, SER188PHE along with VAL255ILE were detected in all four ST149 strains. Mutation GLY186VAL was detected in ST2, ST575, and ST1406, and mutation GLU121LYS was detected in ST10, ST116, and ST623.

It was noted that few isolates of ST575 and ST1406 did not show any overexpression of AdeB and did not possess any mutation in AdeS, in contrast with some isolates of ST575 and ST1406 that did show overexpression of AdeB and mutation GLY186VAL. ST575 and ST1406 are SLVs (Single Locus Variants) of ST10 but did not possess mutations similar to ST10. On the other hand, ST116 and ST623 are SLVs of ST1 but show mutation similar to ST10. Therefore, isolates with similar STs or their SLVs did not possess similar mutational pattern within AdeRS. This emphasizes that not all of these novel mutations are lineage specific (Table 3).

Apart from these mutations associated with overexpression of AdeB, 7 other polymorphisms (ALA94VAL, LEU172PRO, PHE214LEU, TYR303PHE, ILE331VAL, LEU322PHE, and SER341CYS) in AdeS and VAL120ILE and ALA136VAL in AdeR were detected within the isolates, but these mutations were not associated with overexpression of AdeB as they were also present within the reference strains (Table 3). These polymorphisms were also detected in different STs without any lineage-specific distribution (Table 3).

## DISCUSSION

This study addresses two aspects that enhance our understanding of carbapenem resistance in A. baumannii. First, it provides insight into the molecular interaction of different carbapenem residues and EPI PAβN with AdeB transporter at three binding sites (periplasmic, proximal, and distal) using molecular docking. Previously, Su et al. had studied the interaction of the same pump with imipenem only; no other carbapenem interaction was studied (22). The attempt to study all FDA-approved carbapenems along with the inhibitor has not yet been undertaken. Secondly, this study has correlated the novel mutations within the regulator AdeRS with overexpression of AdeABC efflux pump and carbapenem resistance in A. baumannii using predictive modeling-based 3D structure analysis. The previous studies that investigated AdeRS mutations had correlated them with resistance to other antimicrobials, not with carbapenem resistance (31–37). Mutations in AdeS have been reported to affect the phosphorylation activity (31, 32), which is essential for activation of AdeR. The AdeR binds to the ICS region and controls the expression of AdeB. However, the loose binding of AdeR to ICS leads to overexpression of the AdeB efflux pump, resulting in drug resistance (38).

The AdeB efflux pump tends to pump out the carbapenems. Thus, to understand the carbapenem binding with the same, the docking of carbapenems with AdeB has been performed. Docking is a process in the field of *in-silico* modeling study that predicts the favored orientation and optimized interaction of one molecule with a second when a stable complex is formed (here AdeABC pump open and closed conformations and carbapenems/PAβN). Atomic information regarding such interaction is helpful in understanding the basis of complex formation, whereas insight regarding the optimized orientation may be useful to predict the strength of association or predicting binding energy between the two molecules forming the complex. Therefore, the process of molecular docking provides information regarding the nature of the molecule-forming complex as well as the strength of the molecular interactions. In this study, molecular docking showed how FDA-approved carbapenems and PAβN as small molecules were bound in all three probable binding sites (periplasmic entrance, proximal site, and distal hydrophobic patch site) of open conformation of AdeB as a macromolecular target.

The drug enters through the periplasmic or entrance site. Here, due to the presence of substrate or drugs, the AdeB changes from closed extrusion conformation to open binding conformation (15). In the present study, the periplasmic bindings for the open and closed conformers of AdeB were found to be very different, and binding in the closed structure was lower than open conformation. Differences in orientations of three regions were observed (Fig. S5). The β-sheet from region LYS703-GLY715 was shifted outwards in open state to facilitate the entrance of drugs. AcrB residues F664, F666 and R717 were reported to be essential residues in drug recognition (39–41). Their corresponding residues in AdeB are MET656, VAL658, and TRP708, which were also observed to interact with carbapenems in the present study. In the open state, the G-loop (GLY609-ASN618) was tilted toward the periplasmic site and the residue PHE612 interacted with carbapenems. The corresponding residue PHE617 of the AcrB efflux pump was previously reported to have importance in binding (40).

10.1128/msystems.00217-22.5 FIG S5 The orientation of the periplasmic site in the open and closed state of AdeB. The regions W610-A615, K703-G715, and S815-S828 were represented in green, purple, and blue, respectively. Download FIG S5, TIF file, 0.7 MB. Copyright © 2022 Roy et al. 2022 Roy et al. https://creativecommons.org/licenses/by/4.0/ This content is distributed under the terms of the Creative Commons Attribution 4.0 International license.

The proximal site is situated in the inner part of AdeB. It mainly contains the F-loop (PRO661-SER670) and G-loop (GLY609-ASN618) residues (22). The F-loop (flexible loop) has been reported as an important loop in AcrB and in the CusA pump for drug interaction or determination (42, 43). Similarly, in AdeB, an F-loop, ^661^PAIDELGT^668^, was observed where ILE663 was a conserved residue (44). The F-loop is situated in the bottom section of the proximal site. The regions showing different orientation in open and closed conformation of AdeB are listed and presented in Fig. S6. The residues in F-loop ASP664-THR668 were found to be required for substrate recognition in AcrB and residues GLU665-GLY667 are conserved residues in AcrB and MtrD pumps as well (40, 45). Similarly, these residues were found interacting with carbapenems in AdeB. PAβN had also shown interaction with these loop residues. The β-sheet region of ARG813-ILE821 and α-helix of the GLY173-PHE178 region were found shifted outwards to increase the binding domain of the proximal site. A loop near the F-loop with residues ALA33-PRO41 also showed outward movement. Due to the position of another loop, Q128-M138, in the closed structure, docking of carbapenem was observed to be scattered toward the F-loop and G-loop separately. However, in open conformation of AdeB, the binding was at the desired proximal site only.

10.1128/msystems.00217-22.6 FIG S6 The orientation of the proximal site in the open and closed state of AdeB. The regions A33-P41, Q128-M138, G173-F178, A606-N618, V658-S673, and R813-I821 are represented in orange, green, blue, purple, limon, and red, respectively. Download FIG S6, TIF file, 0.8 MB. Copyright © 2022 Roy et al. 2022 Roy et al. https://creativecommons.org/licenses/by/4.0/ This content is distributed under the terms of the Creative Commons Attribution 4.0 International license.

Lastly, the distal site is a hydrophobic patch and has residues PHE178, PHE277, ILE279, ILE607, and TRP610. The G-loop in the distal site of open state was found away from the closed state as it leaned toward the periplasmic domain. The corresponding residues showed the impact of drug binding in AcrB pump (40). This orientation of the G-Loop in closed state makes more space in the distal site to accommodate the substrate. Likewise, β-sheet regions GLN128-MET138, GLY173-PHE178, and GLN273-LEU280 had shown an outward or opening-like shift. Thus, the site could easily accommodate carbapenems (Fig. S7).

10.1128/msystems.00217-22.7 FIG S7 The orientation of the distal site hydrophobic patch in the open and closed state of AdeB. The regions Q128-M138, G173-F178, Q273-L280, and A606-N618 were represented in green, red, blue, and purple, respectively. Download FIG S7, TIF file, 0.7 MB. Copyright © 2022 Roy et al. 2022 Roy et al. https://creativecommons.org/licenses/by/4.0/ This content is distributed under the terms of the Creative Commons Attribution 4.0 International license.

The open state has shown binding at the periplasmic, proximal, and distal sites. Additionally, in the open state, the binding of each carbapenem was found better in periplasmic and proximal sites compared to the distal site. Since the drug enters from the periplasmic site and binds to the proximal site, the binding energies were expected to be low, indicating good binding affinity. Comparatively lesser binding at the distal site, compared to the other two sites, clearly explains why the extrusion of the carbapenems happens through the distal site. In the closed state, the binding of carbapenems was observed to be distorted and away from the binding site (for all three sites) of open state. This has been discussed earlier and can be observed in Fig. 1. Furthermore, the comparison between binding energies of the substrates of AdeB, i.e., carbapenems and PAβN, had shown that the molecular interaction energy of PAβN is much higher than all of the carbapenems. This was observed for all three clefts, which indicates tight binding of PAβN with AdeB. PAβN binds to the carbapenem binding residues, thereby inhibiting the binding of carbapenem to the AdeABC pump. In addition, its double ring blocks the carbapenem interacting residues too and probably generates a steric hindrance, resulting in unavailability of the binding site to other antibiotics. This is in accordance with previous studies that showed PAβN being a competitive inhibitor that binds within the pocket of the efflux pump, where the substrates usually bind, preventing extrusion of several antibiotics. (46–50).

High carbapenem resistance (68%) was observed among the strains collected across 2007-2015. The overexpression of AdeABC efflux pump (29%) was seen in diverse strains (Table 3). Mutations within AdeRS were not found to be lineage specific and the mutations were clearly not due to clonal carbapenem-resistant strains, ensuring a better insight regarding resistance due to pump overexpression. Mutations were detected in AdeRS, more in AdeS, and fewer in AdeR. The three mutations (GLY186VAL, SER188PHE, and GLU121LYS) in the dimerization domain prevented auto-phosphorylation of AdeS, which inhibited the phospho-transfer to AdeR. Hence, AdeR could not bind to the ICS region, resulting in overexpression of AdeABC efflux pump. On the other hand, the mutation VAL255ILE in the catalytic domain of AdeS did not result in any conformatory effect on activity of AdeS. This particular mutation was found in ST149 strains, which also had SER188PHE mutation, and thus these strains showed overexpression of AdeABC efflux pump (Table 5). The other mutations (GLY186VAL, GLU121LYS) were detected among different STs, not linked to any particular sequence type. In the case of AdeR, two substitutions (VAL120ILE and ALA136VAL) were detected across several AdeABC-overexpressing strains, but these were considered as silent polymorphisms as these mutations had no significant effect on the structure of AdeR. GLY186VAL, found here, is the only mutation that has been reported earlier in a tigecycline-resistant A. baumannii (35), tigecycline being an antibiotic that is extruded through the AdeABC pump. Some mutations in AdeS (GLY103ASP, THR153MET, ARG152LYS, ASN125LYS, GLY336SER, HIS189TYR, and ILE252SER) and AdeR (PRO56SER, ALA91VAL, PRO116LEU, LEU192ARG, and GLU219ALA) reported earlier to be associated with overexpression of AdeABC and resistance to other antibiotics, were not detected in this study (26, 51). Furthermore, disruption of *adeRS* by insertion of IS elements and nucleotide deletions or insertion were not evident in this study, as reported in other earlier studies (34, 36, 52). Four (A_130, A_133, A_135, and A_169) of the 15 CRABs that showed reduction of MIC for carbapenems in the presence of PAβN, did not show overexpression of AdeABC pump. Apart from AdeABC, PAβN is also reported to inhibit the activity of other RND efflux pumps in A. baumannii such as AdeFGH or AdeIJ; thus, involvement of other RND efflux pumps in carbapenem resistance in these four isolates cannot be ruled out (34). Furthermore, PAβN permeabilizes both inner and outer membranes by which it gains entry into the cells where it can access the efflux pump targets (53).

Despite the recognized role of the AdeABC efflux system in carbapenem resistance (27–30), in-depth analysis of the contribution of this pump and its regulators still remains challenging due to the lack of data. This shows the urgent need of efflux pump-associated resistance surveillance in A. baumannii so that steps can be taken to find better alternative therapies against these recalcitrant organisms. There are limited experimental studies that elucidate the role of certain carbapenems to AdeABC pump (24, 25). Hence, the use of *in silico* methods to study complex structures of transporters (AdeB), their regulators, and their molecular interactions with their substrates such as carbapenems and PAβN can add insight about fundamental mechanisms of resistance development. Recognition of the critical residues of AdeB that interact with both carbapenems and PAβN could contribute to the design of new effective and selective EPIs that may play key roles in reversing antimicrobial resistance.

## MATERIALS AND METHODS

### Docking of carbapenems with AdeABC efflux pump.

The structures of all FDA approved carbapenems (imipenem, meropenem, ertapenem, doripenem, biapenem, tebipenem) were generated using ChemDraw. ChemDraw 2D was used for sketching two-dimensional structures, and then they were exported to Chem3D to make three-dimensional structures. The energy minimization was performed using the MM2 minimization tool present in Chem3D. Resultant structures were used to make pdbqt files for docking preparation for the ligand. The cryo-EM structure of AdeB from A. baumannii with PDB IDs 7KGD and 7KGG were used as an open state and closed state of AdeB, respectively (26). The ligands (carbapenems and PAβN) and enzyme (AdeB) pdbqt file generation was performed using AutoDock Tools 1.5.6. AdeB has three potential binding sites reported, i.e., entrance or periplasmic site, proximal site (F-loop and G-loop), and distal site (hydrophobic patch) of AdeB from A. baumannii (22, 26). Hence, the grid was set for all three sites separately. For the periplasmic site, the center of the grid was set at(196.76, 149.313, 166.905) Å and the dimension of the box was 22 × 18×18 Å^3^. In the case of the proximal site, the grid box was centered at 189.375, 159.042, 166.614 Å with dimensions 22 × 22×30 Å^3^. Lastly, for the distal site, the grid center was 182.605, 163.692, 157.425 Å and the grid dimension was 20 × 22×20 Å^3^. The default Vina grid spacing was 1 Å. The docking was carried out for 10 conformations using AutoDockVina. The lowest binding energy structures were extracted from the result, and the complexes were prepared. The interaction visualization and figure formation were carried out by PyMOL (http://www.pymol.org). The MAT tool (http://hazralab.iitr.ac.in/) was used for getting information about contacts within 4 Å of the ligand (carbapenems and PAβN) enzyme (AdeB).

### Carbapenem susceptibility, MLST and efflux pump inhibitor test.

A. baumannii included in the study were isolated from the blood of septicemic neonates admitted to the NICU of IPGMER and SSKM hospital, Kolkata, India, during 2007–2015. Strains were identified initially by the Vitek 2 compact system (bioMérieux, Marcy l’Etoile, France). Further confirmation was done by detection of *bla*_OXA-51_. MLST was carried out using specific primers of 7 housekeeping genes (*cnp60*, *fusA*, *gltA*, *pyrG*, *recA*, *rplB*, and *rpoB*) and conditions described in the A. baumannii MLST Pasteur Scheme (https://pubmlst.org/abaumannii/). The allele numbers are combined to yield a specific ST using the pubMLST database.

The MIC values (mg/L) of two carbapenems (meropenem and imipenem) (Sigma-Aldrich, St. Louis, MO, USA) were determined using broth microdilution method and interpreted according to the CLSI (Clinical and Laboratory Standards Institute) breakpoints for Acinetobacter spp. (meropenem and imipenem: susceptible ≤2 mg/L; resistant ≥8 mg/L) (54). The MIC_90_ values for carbapenems were also calculated. To assess whether carbapenem resistance is reversible with an efflux pump inhibitor, MIC of meropenem and imipenem was determined with and without EPI PAβN (Sigma) at a concentration of 50 mg/L. A significant inhibition was defined as a 4-fold or greater reduction of MIC in the presence of PAβN (55).

### Study of the overexpression of AdeABC efflux pump by quantitative reverse transcriptase PCR.

CRABs showing ≥4-fold reduction of MIC in the presence of PAβN (for meropenem and/or imipenem) were considered for qRT-PCR to check the expression level of AdeABC efflux pump. Since inner membrane proteins are the most crucial part of tripartite RND pumps, expression of *adeB* was evaluated using primers as described previously (56). Total RNA (2 μg) was isolated from CRABs using a Nucleospin RNA isolation kit (Nucleospin, Macherey-Nagel, Düren, Germany), and cDNA was synthesized with a high-capacity cDNA reverse transcription kit (Applied Biosystems, Warrington, United Kimgdom). The relative gene expression (Δ*C_T_*) for the pump gene transcript was calculated against that for the 16S rRNA gene, and the △△CT was calculated against that for the susceptible strain A. baumannii ATCC 19606 (expression = 1), which served as the control. Relative expression level of pump genes was calculated by the 2^−△△CT^ method. An effect on gene expression was considered significant when the corresponding ratios were >2.0.

### Nucleotide sequencing of AdeRS.

The regulator (AdeRS) of AdeABC pump was sequenced for CRABs, which showed overexpression of the *adeB* gene. Sequencing of *adeR* and *adeS* genes was carried out as previously described (57, 58). Protein sequences of AdeRS were compared with four reference strains including A. baumannii ATCC 19606, A. baumannii ATCC 17978, A. baumannii AYE, and A. baumannii ACICU in order to exclude polymorphisms.

### Structural modeling of AdeRS.

The AdeS sequence was used to perform BLAST against the PDB database to search for a template. The histidine kinase dimeric structure with PDB ID 4BIU was selected as a template because it had 30% identity and 91% query coverage. The structure was modeled using the SWISS-MODEL online Web server. In the case of AdeR modeling, two templates were used. The receiver domain template was the protein structure with PDB ID 5X5L, and for the DNA binding domain, the structure with PDB ID 5X5J was considered. The AdeR has a connecting loop between these two domains. Thus, Modeller was used to perform modeling of the AdeR sequence. Additionally, the structure with PDB ID 6OWS was taken for the study of the efflux pump of A. baumannii (AdeB). Furthermore, the mutations detected in the study strains (by sequencing) were incorporated by using the Simple Mutation tool of COOT (http://journals.iucr.org/d/issues/2004/12/01/isscontsbdy.html) and analyzed.

### Energy minimization of structures.

The energy minimization was performed for all the structures using GROMACS v2018.1 software on the Linux platform. During the energy minimization, AMBER ff99sb-ILDN was used with the TIP3P water model. The dodecahedron box with 1 nm width was created for the system preparation in GROMACS, and required sodium or chlorine ions were added to neutralize the system. The energy minimization was performed using the steepest descent algorithm and followed by the conjugate gradient method. The maximum force cutoff of 1,000.00 kJ/mol/nm was used, and the Verlet method was used for buffered neighbor searching. The structural visualization and modifications were performed using PyMOL and COOT. The dihedral distribution-based validation of mutated structures was performed by PROCHECK.

### DNA docking with AdeR.

The AdeR was found to interact with the ICS region. Thus, to analyze the interactions, docking of AdeR with DNA was performed using HDOCK. The DNA structure was extracted from 5X5L, and then energy was minimized using the same energy minimization technique mentioned above. The DNA binding site information was extracted from a docking template with PDB id 5X5L. The residues of the binding site were specified for both receptor (i.e., AdeR) and ligand (i.e., DNA). After docking, the complex structure mimicking the template pose was selected for further analysis.
