# Supplementary material for: Differential Binding of Carbapenems with the AdeABC Efflux Pump and Modulation of the Expression of AdeB Linked to Novel Mutations within Two-Component System AdeRS in Carbapenem-Resistant Acinetobacter baumannii
Source: mSystems. 2022 Jun 23;7(4):e00217-22. doi: 10.1128/msystems.00217-22 (PMC9426577; doi:10.1128/msystems.00217-22)
Supplement: TABLE S1 [file msystems.00217-22-s0008.docx]

**Table S1.**

1. **Periplasmic site**

| **Close-conf--Biapenem** | | | | | | |
| --- | --- | --- | --- | --- | --- | --- |
| Ligand | Atom_Name |  | Residue_Name | Residue_No | Atom_Name | Distance |
| Biapenem | N3 |  | SER | 641 | OG | 3.1 |
| Biapenem | O21 |  | LEU | 659 | N | 3.6 |
| Biapenem | O23 |  | GLU | 563 | OE1 | 3.9 |
| Biapenem | O24 |  | ASN | 644 | ND2 | 3.8 |
| **Close-conf--Doripenem** | | | | | | |
| Ligand | Atom_Name |  | Residue_Name | Residue_No | Atom_Name | Distance |
| Doripenem | N4 |  | TYR | 705 | O | 3.1 |
| Doripenem | O21 |  | GLU | 654 | OE1 | 3.9 |
| Doripenem | O23 |  | VAL | 707 | N | 3.5 |
| **Close-conf--Ertapenem** | | | | | | |
| Ertapenem | Atom_Name |  | Residue_Name | Residue_No | Atom_Name | Distance |
| Ertapenem | N2 |  | TYR | 705 | O | 3.6 |
| Ertapenem | O32 |  | MET | 706 | N | 3.7 |
| Ertapenem | O32 |  | PHE | 704 | O | 3.1 |
| Ertapenem | O35 |  | MET | 695 | O | 3.9 |
| **Close-conf--Imipenem** | | | | | | |
| Ligand | Atom_Name |  | Residue_Name | Residue_No | Atom_Name | Distance |
| Imipenem | N2 |  | SER | 641 | OG | 3.2 |
| Imipenem | N3 |  | LYS | 638 | N | 3.4 |
| Imipenem | N3 |  | SER | 637 | O | 3.2 |
| Imipenem | N3 |  | SER | 637 | OG | 3.0 |
| Imipenem | O16 |  | LEU | 659 | N | 3.9 |
| Imipenem | O19 |  | ASN | 644 | ND2 | 3.8 |
| **Close-conf--Meropenem** | | | | | | |
| Ligand | Atom_Name |  | Residue_Name | Residue_No | Atom_Name | Distance |
| Meropenem | N2 |  | TYR | 705 | O | 3.4 |
| Meropenem | N3 |  | ALA | 698 | O | 3.9 |
| Meropenem | O22 |  | GLU | 654 | OE1 | 3.7 |
| Meropenem | O26 |  | GLN | 574 | NE2 | 3.8 |
| **Close-conf--Tebipenem** | | | | | | |
| Ligand | Atom_Name |  | Residue_Name | Residue_No | Atom_Name | Distance |
| Tebipenem | N2 |  | GLU | 654 | OE2 | 3.6 |
| **Open-conf--Biapenem** | | | | | | |
| Ligand | Atom_Name |  | Residue_Name | Residue_No | Atom_Name | Distance |
| Biapenem | O22 |  | SER | 613 | OG | 3.8 |
| Biapenem | O22 |  | GLY | 711 | N | 3.9 |
| Biapenem | O23 |  | GLU | 710 | N | 3.9 |
| Biapenem | O24 |  | MET | 656 | SD | 3.7 |
| Biapenem | O24 |  | MET | 656 | O | 3.9 |
| Biapenem | S |  | GLN | 574 | NE2 | 3.7 |
| **Open-conf--Doripenem** | | | | | | |
| Ligand | Atom_Name |  | Residue_Name | Residue_No | Atom_Name | Distance |
| Doripenem | N2 |  | LEU | 659 | O | 3.1 |
| Doripenem | N3 |  | LEU | 659 | N | 2.9 |
| Doripenem | N3 |  | GLU | 563 | OE1 | 3.8 |
| Doripenem | N4 |  | ALA | 657 | O | 3.8 |
| Doripenem | O21 |  | GLY | 820 | O | 3.8 |
| Doripenem | O25 |  | THR | 668 | N | 2.8 |
| Doripenem | O26 |  | GLY | 820 | N | 3.2 |
| **Open-conf--Ertapenem** | | | | | | |
| Ligand | Atom_Name |  | Residue_Name | Residue_No | Atom_Name | Distance |
| Ertapenem | N1 |  | GLU | 710 | OE2 | 3.0 |
| Ertapenem | O29 |  | GLY | 711 | O | 3.1 |
| Ertapenem | O29 |  | GLY | 711 | N | 3.0 |
| Ertapenem | O29 |  | SER | 613 | OG | 3.5 |
| Ertapenem | O30 |  | PHE | 612 | N | 3.1 |
| Ertapenem | O31 |  | TRP | 708 | NE1 | 3.3 |
| Ertapenem | O32 |  | GLY | 671 | O | 3.9 |
| Ertapenem | O32 |  | SER | 673 | OG | 3.5 |
| Ertapenem | O33 |  | GLY | 820 | N | 2.9 |
| **Open-conf--Imipenem** | | | | | | |
| Ligand | Atom_Name |  | Residue_Name | Residue_No | Atom_Name | Distance |
| Imipenem | N1 |  | SER | 572 | OG | 3.9 |
| Imipenem | N2 |  | SER | 613 | OG | 3.7 |
| Imipenem | N2 |  | GLY | 711 | N | 3.1 |
| Imipenem | N2 |  | ASN | 709 | O | 2.9 |
| Imipenem | N2 |  | GLY | 711 | O | 3.0 |
| Imipenem | N3 |  | GLN | 574 | NE2 | 3.9 |
| Imipenem | O16 |  | PHE | 573 | N | 3.9 |
| Imipenem | O16 |  | GLN | 574 | N | 3.6 |
| Imipenem | O18 |  | MET | 656 | SD | 3.6 |
| **Open-conf--Meropenem** | | | | | | |
| Ligand | Atom_Name |  | Residue_Name | Residue_No | Atom_Name | Distance |
| Meropenem | N2 |  | TRP | 708 | NE1 | 3.4 |
| Meropenem | O22 |  | GLN | 574 | N | 3.9 |
| Meropenem | O26 |  | SER | 613 | N | 3.0 |
| **Open-conf--Tebipenem** | | | | | | |
| Ligand | Atom_Name |  | Residue_Name | Residue_No | Atom_Name | Distance |
| Tebipenem | N3 |  | LEU | 659 | O | 3.8 |
| Tebipenem | O27 |  | GLU | 710 | OE2 | 3.6 |
| Tebipenem | O32 |  | SER | 673 | N | 3.8 |

1. **Proximal site**

| **Close-conf--Biapenem** | | | | | | |
| --- | --- | --- | --- | --- | --- | --- |
| Ligand | Atom_Name |  | Residue_Name | Residue_No | Atom_Name | Distance |
| Biapenem | N1 |  | GLU | 89 | OE1 | 3.2 |
| Biapenem | N1 |  | PHE | 612 | O | 3.4 |
| Biapenem | N2 |  | ALA | 80 | O | 3.8 |
| Biapenem | N2 |  | SER | 79 | OG | 3.2 |
| Biapenem | N4 |  | ARG | 675 | NH1 | 3.8 |
| Biapenem | N4 |  | ARG | 675 | NE | 3.6 |
| Biapenem | O22 |  | SER | 613 | N | 3.6 |
| Biapenem | O22 |  | GLY | 611 | O | 3.1 |
| **Close-conf--Doripenem** | | | | | | |
| Ligand | Atom_Name |  | Residue_Name | Residue_No | Atom_Name | Distance |
| Doripenem | N2 |  | GLU | 710 | OE1 | 3.7 |
| Doripenem | N2 |  | THR | 668 | OG1 | 3.7 |
| Doripenem | N2 |  | GLU | 710 | OE2 | 3.2 |
| Doripenem | N3 |  | PHE | 612 | O | 3.9 |
| Doripenem | N3 |  | SER | 613 | OG | 3.9 |
| Doripenem | N3 |  | SER | 613 | N | 3.7 |
| Doripenem | N4 |  | PHE | 612 | N | 3.2 |
| Doripenem | O20 |  | ALA | 80 | O | 3.2 |
| Doripenem | O20 |  | SER | 79 | OG | 3.4 |
| Doripenem | O20 |  | ALA | 80 | N | 3.7 |
| Doripenem | O20 |  | THR | 81 | N | 3.9 |
| Doripenem | O24 |  | SER | 817 | OG | 3.0 |
| Doripenem | O25 |  | GLU | 89 | O | 3.2 |
| **Close-conf--Ertapenem** | | | | | | |
| Ligand | Atom_Name |  | Residue_Name | Residue_No | Atom_Name | Distance |
| Ertapenem | N1 |  | THR | 668 | OG1 | 2.8 |
| Ertapenem | O26 |  | ALA | 80 | N | 3.5 |
| Ertapenem | O26 |  | THR | 81 | N | 3.8 |
| Ertapenem | O26 |  | GLU | 89 | O | 3.9 |
| Ertapenem | O29 |  | ARG | 675 | NH1 | 3.3 |
| Ertapenem | O30 |  | GLU | 710 | OE1 | 3.4 |
| Ertapenem | O30 |  | GLU | 710 | OE2 | 3.5 |
| Ertapenem | O32 |  | GLY | 852 | N | 3.2 |
| Ertapenem | O32 |  | THR | 851 | OG1 | 2.8 |
| **Close-conf--Imipenem** | | | | | | |
| Ligand | Atom_Name |  | Residue_Name | Residue_No | Atom_Name | Distance |
| Imipenem | N1 |  | SER | 613 | OG | 3.3 |
| Imipenem | N2 |  | SER | 572 | OG | 3.1 |
| Imipenem | N3 |  | MET | 656 | O | 3.1 |
| Imipenem | O16 |  | PHE | 573 | N | 3.9 |
| Imipenem | O16 |  | GLN | 574 | OE1 | 3.9 |
| Imipenem | O19 |  | TRP | 610 | O | 3.9 |
| Imipenem | O19 |  | ALA | 620 | N | 2.8 |
| **Close-conf--Meropenem** | | | | | | |
| Ligand | Atom_Name |  | Residue_Name | Residue_No | Atom_Name | Distance |
| Meropenem | N1 |  | GLU | 89 | OE1 | 3.3 |
| Meropenem | N1 |  | PHE | 612 | O | 3.5 |
| Meropenem | N2 |  | SER | 79 | OG | 2.8 |
| Meropenem | N2 |  | THR | 91 | OG1 | 3.8 |
| Meropenem | O24 |  | SER | 613 | N | 3.7 |
| Meropenem | O24 |  | GLY | 611 | O | 3.0 |
| **Close-conf--Tebipenem** | | | | | | |
| Ligand | Atom_Name |  | Residue_Name | Residue_No | Atom_Name | Distance |
| Tebipenem | N2 |  | GLU | 89 | OE1 | 3.9 |
| Tebipenem | N2 |  | SER | 134 | OG | 3.6 |
| Tebipenem | N3 |  | PHE | 612 | O | 3.3 |
| Tebipenem | O26 |  | THR | 851 | OG1 | 3.4 |
| Tebipenem | O26 |  | GLY | 852 | N | 3.9 |
| Tebipenem | O26 |  | TYR | 77 | OH | 3.6 |
| **Open-conf--Biapenem** | | | | | | |
| Ligand | Atom_Name |  | Residue_Name | Residue_No | Atom_Name | Distance |
| Biapenem | N2 |  | ASP | 664 | OD2 | 3.4 |
| Biapenem | O21 |  | ALA | 662 | O | 3.2 |
| Biapenem | O23 |  | PHE | 136 | N | 3.3 |
| **Open-conf--Doripenem** | | | | | | |
| Ligand | Atom_Name |  | Residue_Name | Residue_No | Atom_Name | Distance |
| Doripenem | N2 |  | PHE | 136 | O | 2.9 |
| Doripenem | N2 |  | THR | 329 | OG1 | 3.6 |
| Doripenem | N3 |  | PHE | 35 | N | 3.3 |
| Doripenem | N3 |  | ARG | 34 | N | 3.0 |
| Doripenem | N3 |  | PHE | 35 | O | 3.9 |
| Doripenem | N4 |  | PHE | 136 | N | 3.9 |
| Doripenem | N4 |  | SER | 134 | O | 2.7 |
| Doripenem | O23 |  | ASP | 664 | OD1 | 3.9 |
| Doripenem | O23 |  | ILE | 663 | O | 3.5 |
| Doripenem | S26 |  | ALA | 662 | O | 3.7 |
| **Open-conf--Ertapenem** | | | | | | |
| Ligand | Atom_Name |  | Residue_Name | Residue_No | Atom_Name | Distance |
| Ertapenem | O28 |  | ASP | 664 | OD2 | 3.2 |
| Ertapenem | O28 |  | ILE | 663 | O | 3.2 |
| Ertapenem | O29 |  | PHE | 136 | N | 3.2 |
| Ertapenem | O32 |  | GLN | 176 | OE1 | 3.1 |
| **Open-conf--Imipenem** | | | | | | |
| Ligand | Atom_Name |  | Residue_Name | Residue_No | Atom_Name | Distance |
| Imipenem | N2 |  | TYR | 327 | OH | 3.1 |
| Imipenem | N3 |  | ARG | 34 | NH2 | 3.8 |
| Imipenem | N3 |  | ALA | 662 | O | 2.9 |
| Imipenem | N3 |  | GLN | 566 | OE1 | 3.1 |
| Imipenem | N3 |  | GLN | 566 | NE2 | 3.7 |
| Imipenem | N3 |  | PRO | 661 | O | 3.0 |
| Imipenem | O19 |  | THR | 329 | OG1 | 2.9 |
| **Open-conf--Meropenem** | | | | | | |
| Ligand | Atom_Name |  | Residue_Name | Residue_No | Atom_Name | Distance |
| Meropenem | N1 |  | SER | 134 | O | 3.9 |
| Meropenem | N2 |  | TYR | 327 | OH | 3.2 |
| Meropenem | O21 |  | PHE | 35 | O | 3.2 |
| Meropenem | O23 |  | ILE | 663 | O | 3.4 |
| Meropenem | O24 |  | SER | 37 | N | 3.3 |
| Meropenem | O25 |  | THR | 329 | OG1 | 3.0 |
| Meropenem | S26 |  | ALA | 662 | O | 3.9 |
| Meropenem | S26 |  | ASP | 664 | OD2 | 3.9 |
| **Open-conf--Tebipenem** | | | | | | |
| Ligand | Atom_Name |  | Residue_Name | Residue_No | Atom_Name | Distance |
| Tebipenem | N2 |  | GLU | 665 | OE2 | 3.7 |
| Tebipenem | N2 |  | ASP | 664 | OD2 | 3.6 |
| Tebipenem | N3 |  | ASP | 664 | OD1 | 3.7 |
| Tebipenem | O26 |  | THR | 329 | OG1 | 3.6 |
| Tebipenem | O27 |  | ARG | 34 | NH2 | 3.7 |
| Tebipenem | S33 |  | SER | 133 | O | 3.7 |

1. **Distal site**

| **Close-conf--Biapenem** | | | | | | |
| --- | --- | --- | --- | --- | --- | --- |
| Ligand | Atom_Name |  | Residue_Name | Residue_No | Atom_Name | Distance |
| Biapenem | N2 |  | SER | 134 | OG | 3.9 |
| Biapenem | N3 |  | GLN | 292 | OE1 | 3.7 |
| Biapenem | N3 |  | GLN | 176 | OE1 | 3.8 |
| Biapenem | O20 |  | GLU | 130 | OE1 | 3.1 |
| Biapenem | O20 |  | GLU | 130 | OE2 | 3.9 |
| Biapenem | O22 |  | GLU | 130 | O | 3.0 |
| **Close-conf--Doripenem** | | | | | | |
| Ligand | Atom_Name |  | Residue_Name | Residue_No | Atom_Name | Distance |
| Doripenem | N1 |  | SER | 134 | OG | 3.3 |
| Doripenem | N1 |  | ASN | 44 | ND2 | 3.6 |
| Doripenem | N1 |  | GLU | 89 | OE1 | 3.9 |
| Doripenem | N2 |  | GLU | 89 | OE2 | 3.3 |
| Doripenem | N3 |  | ASN | 44 | OD1 | 3.2 |
| Doripenem | N4 |  | GLU | 130 | N | 3.4 |
| Doripenem | N4 |  | GLU | 130 | O | 3.4 |
| Doripenem | N4 |  | ASN | 44 | O | 2.9 |
| Doripenem | N4 |  | GLU | 130 | OE1 | 2.8 |
| Doripenem | O24 |  | SER | 133 | O | 3.8 |
| Doripenem | O24 |  | SER | 134 | N | 3.3 |
| Doripenem | O24 |  | SER | 132 | O | 3.7 |
| **Close-conf--Ertapenem** | | | | | | |
| Ligand | Atom_Name |  | Residue_Name | Residue_No | Atom_Name | Distance |
| Ertapenem | N1 |  | ASN | 44 | OD1 | 3.9 |
| Ertapenem | N1 |  | GLU | 130 | OE1 | 3.8 |
| Ertapenem | O32 |  | GLN | 292 | OE1 | 2.9 |
| Ertapenem | S33 |  | GLN | 176 | NE2 | 3.7 |
| **Close-conf--Imipenem** | | | | | | |
| Ligand | Atom_Name |  | Residue_Name | Residue_No | Atom_Name | Distance |
| Imipenem | N1 |  | ASN | 44 | ND2 | 3.8 |
| Imipenem | N1 |  | SER | 132 | OG | 3.8 |
| Imipenem | N2 |  | SER | 134 | OG | 3.9 |
| Imipenem | N2 |  | GLY | 135 | N | 3.9 |
| Imipenem | N3 |  | GLN | 292 | OE1 | 2.9 |
| Imipenem | N3 |  | GLN | 176 | OE1 | 2.9 |
| Imipenem | O16 |  | GLU | 130 | O | 3.5 |
| Imipenem | O17 |  | SER | 132 | O | 3.6 |
| Imipenem | O18 |  | SER | 134 | N | 3.3 |
| Imipenem | O18 |  | SER | 133 | O | 3.7 |
| **Close-conf--Meropenem** | | | | | | |
| Ligand | Atom_Name |  | Residue_Name | Residue_No | Atom_Name | Distance |
| Meropenem | N1 |  | LYS | 174 | NZ | 3.9 |
| Meropenem | N2 |  | PHE | 612 | O | 2.8 |
| Meropenem | N3 |  | SER | 134 | OG | 3.4 |
| Meropenem | O21 |  | ASN | 44 | OD1 | 3.8 |
| Meropenem | O21 |  | ASN | 44 | ND2 | 3.9 |
| Meropenem | O23 |  | SER | 133 | O | 3.6 |
| Meropenem | O23 |  | GLN | 292 | NE2 | 3.9 |
| Meropenem | S26 |  | GLN | 176 | OE1 | 3.8 |
| **Close-conf--Tebipenem** | | | | | | |
| Ligand | Atom_Name |  | Residue_Name | Residue_No | Atom_Name | Distance |
| Tebipenem | O28 |  | GLU | 89 | OE2 | 3.9 |
| Tebipenem | O30 |  | LYS | 174 | NZ | 3.9 |
| Tebipenem | S32 |  | THR | 87 | OG1 | 3.9 |
| **Open-conf--Biapenem** | | | | | | |
| Ligand | Atom_Name |  | Residue_Name | Residue_No | Atom_Name | Distance |
| Biapenem | N1 |  | TRP | 610 | NE1 | 3.6 |
| Biapenem | N2 |  | ASP | 83 | OD2 | 3.8 |
| Biapenem | N4 |  | THR | 87 | O | 3.4 |
| **Open-conf--Doripenem** | | | | | | |
| Ligand | Atom_Name |  | Residue_Name | Residue_No | Atom_Name | Distance |
| Doripenem | N1 |  | TRP | 610 | NE1 | 3.7 |
| Doripenem | N3 |  | ALA | 615 | N | 3.9 |
| Doripenem | N3 |  | ASP | 83 | OD2 | 3.0 |
| Doripenem | N4 |  | PHE | 612 | O | 3.3 |
| Doripenem | N4 |  | GLY | 614 | N | 3.2 |
| Doripenem | N4 |  | GLY | 614 | O | 3.6 |
| Doripenem | N4 |  | GLY | 611 | O | 2.8 |
| **Open-conf--Ertapenem** | | | | | | |
| Ligand | Atom_Name |  | Residue_Name | Residue_No | Atom_Name | Distance |
| Ertapenem | O26 |  | GLN | 273 | NE2 | 3.8 |
| Ertapenem | O26 |  | GLN | 273 | OE1 | 3.5 |
| Ertapenem | O27 |  | GLN | 128 | NE2 | 3.6 |
| Ertapenem | O27 |  | GLN | 128 | OE1 | 3.3 |
| Ertapenem | S33 |  | THR | 87 | OG1 | 3.9 |
| **Open-conf--Imipenem** | | | | | | |
| Ligand | Atom_Name |  | Residue_Name | Residue_No | Atom_Name | Distance |
| Imipenem | N2 |  | PHE | 612 | O | 3.2 |
| Imipenem | N2 |  | GLY | 611 | O | 3.0 |
| Imipenem | N2 |  | TRP | 610 | NE1 | 3.0 |
| Imipenem | O16 |  | THR | 87 | OG1 | 3.7 |
| Imipenem | O17 |  | THR | 87 | O | 3.3 |
| Imipenem | O17 |  | ASP | 83 | OD2 | 3.4 |
| **Open-conf--Meropenem** | | | | | | |
| Ligand | Atom_Name |  | Residue_Name | Residue_No | Atom_Name | Distance |
| Meropenem | N1 |  | TRP | 610 | NE1 | 3.9 |
| Meropenem | N2 |  | ASN | 44 | ND2 | 3.9 |
| Meropenem | N2 |  | GLU | 89 | OE2 | 3.1 |
| Meropenem | N2 |  | ASN | 44 | OD1 | 3.1 |
| Meropenem | O21 |  | ALA | 615 | N | 3.7 |
| Meropenem | O21 |  | GLY | 614 | N | 3.9 |
| Meropenem | O24 |  | GLY | 611 | O | 3.1 |
| **Open-conf--Tebipenem** | | | | | | |
| Ligand | Atom_Name |  | Residue_Name | Residue_No | Atom_Name | Distance |
| Tebipenem | O31 |  | GLU | 181 | N | 3.9 |
