# Supplementary material for: Differential Binding of Carbapenems with the AdeABC Efflux Pump and Modulation of the Expression of AdeB Linked to Novel Mutations within Two-Component System AdeRS in Carbapenem-Resistant Acinetobacter baumannii
Source: mSystems. 2022 Jun 23;7(4):e00217-22. doi: 10.1128/msystems.00217-22 (PMC9426577; doi:10.1128/msystems.00217-22)
Supplement: TABLE S2 [file msystems.00217-22-s0009.docx]

**Table S2.**

1. **Periplasmic site**

| **Close-conf--PAβN** | | | | | | |
| --- | --- | --- | --- | --- | --- | --- |
| Ligand | Atom_Name |  | Residue_Name | Residue_No | Atom_Name | Distance |
| PAβN | N1 |  | TYR | 705 | O | 3.5 |
| PAβN | N2 |  | VAL | 707 | N | 3.2 |
| PAβN | N2 |  | MET | 695 | SD | 3.8 |
| PAβN | N2 |  | ALA | 698 | O | 3.9 |
| PAβN | N2 |  | ALA | 699 | N | 3.8 |
| PAβN | N3 |  | MET | 706 | N | 3.9 |
| PAβN | N3 |  | PHE | 704 | O | 2.9 |
| PAβN | N6 |  | ASN | 644 | OD1 | 3.2 |
| **Open-conf--PAβN** | | | | | | |
| Ligand | Atom_Name |  | Residue_Name | Residue_No | Atom_Name | Distance |
| PAβN | N1 |  | THR | 668 | OG1 | 3.1 |
| PAβN | N1 |  | THR | 668 | N | 3.4 |
| PAβN | N2 |  | LEU | 659 | O | 3.3 |
| PAβN | N2 |  | GLU | 563 | OE1 | 3.3 |
| PAβN | N3 |  | PRO | 661 | N | 3.5 |
| PAβN | N6 |  | LEU | 659 | N | 3.4 |

1. **Proximal site**

| **Close-conf-- PAβN** | | | | | | |
| --- | --- | --- | --- | --- | --- | --- |
| Ligand | Atom_Name |  | Residue_Name | Residue_No | Atom_Name | Distance |
| PAβN | N1 |  | THR | 851 | OG1 | 3.4 |
| PAβN | N1 |  | TYR | 77 | OH | 2.9 |
| PAβN | N2 |  | GLY | 852 | N | 3.9 |
| PAβN | N2 |  | LEU | 666 | O | 3.0 |
| PAβN | N3 |  | SER | 673 | OG | 3.9 |
| PAβN | N5 |  | SER | 79 | OG | 2.9 |
| PAβN | N5 |  | THR | 91 | OG1 | 3.8 |
| PAβN | N6 |  | ALA | 80 | O | 3.2 |
| PAβN | N6 |  | ALA | 80 | N | 3.3 |
| PAβN | N6 |  | GLU | 89 | O | 3.7 |
| PAβN | N6 |  | THR | 81 | N | 3.8 |
| **Open-conf-- PAβN** | | | | | | |
| Ligand | Atom_Name |  | Residue_Name | Residue_No | Atom_Name | Distance |
| PAβN | N1 |  | SER | 134 | O | 3.9 |
| PAβN | N2 |  | ILE | 663 | O | 3.0 |
| PAβN | N3 |  | GLU | 665 | OE2 | 3.1 |
| PAβN | N3 |  | GLU | 665 | OE1 | 3.4 |
| PAβN | N3 |  | ASP | 664 | OD2 | 3.4 |
| PAβN | N3 |  | ASP | 664 | OD1 | 3.1 |
| PAβN | N6 |  | ARG | 34 | NH2 | 3.4 |
| PAβN | N6 |  | ALA | 662 | O | 3.1 |
| PAβN | N6 |  | TYR | 327 | OH | 3.9 |
| PAβN | N6 |  | GLN | 566 | OE1 | 3.4 |
| PAβN | N6 |  | PRO | 661 | O | 3.9 |

1. **Distal Site**

| **Close-conf--PAβN** | | | | | | |
| --- | --- | --- | --- | --- | --- | --- |
| Ligand | Atom_Name |  | Residue_Name | Residue_No | Atom_Name | Distance |
| PAβN | N1 |  | TRP | 610 | NE1 | 3.3 |
| PAβN | N1 |  | GLU | 89 | OE2 | 3.7 |
| PAβN | N2 |  | ALA | 615 | N | 3.9 |
| PAβN | N2 |  | GLY | 614 | N | 3.6 |
| PAβN | N2 |  | GLY | 614 | O | 3.6 |
| PAβN | N2 |  | GLY | 611 | O | 2.9 |
| PAβN | N3 |  | ASP | 83 | OD2 | 3.2 |
| PAβN | N6 |  | SER | 46 | OG | 3.9 |
| PAβN | O33 |  | THR | 87 | OG1 | 3.9 |
| **Open-conf--PAβN** | | | | | | |
| Ligand | Atom_Name |  | Residue_Name | Residue_No | Atom_Name | Distance |
| PAβN | N1 |  | TRP | 610 | NE1 | 3.2 |
| PAβN | N2 |  | GLY | 611 | O | 3.1 |
| PAβN | N2 |  | PHE | 612 | O | 2.9 |
| PAβN | N2 |  | GLY | 614 | N | 3.9 |

**Figure 8**

**Figure 8**
