# Supplementary figures and images for: Differential Binding of Carbapenems with the AdeABC Efflux Pump and Modulation of the Expression of AdeB Linked to Novel Mutations within Two-Component System AdeRS in Carbapenem-Resistant Acinetobacter baumannii
Source: mSystems. 2022 Jun 23;7(4):e00217-22. doi: 10.1128/msystems.00217-22 (PMC9426577; doi:10.1128/msystems.00217-22)

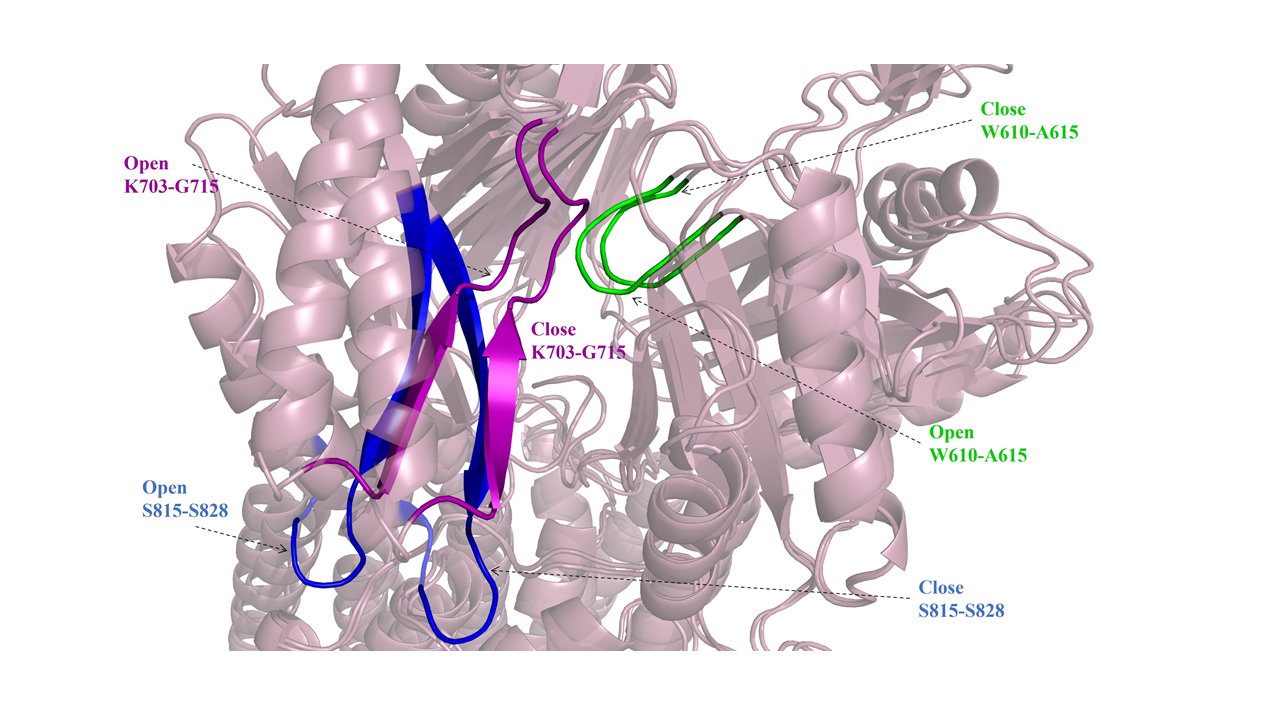

Supplement: FIG S5 [file msystems.00217-22-s0005.tif]

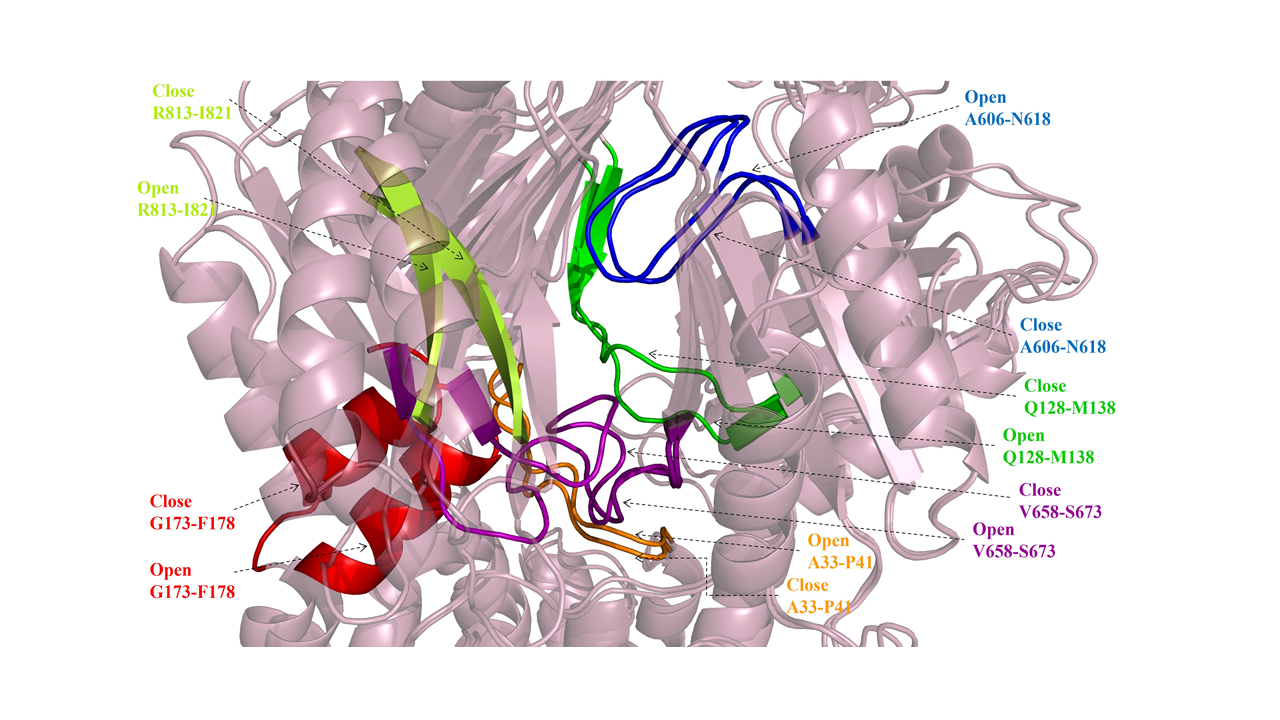

Supplement: FIG S6 [file msystems.00217-22-s0006.tif]

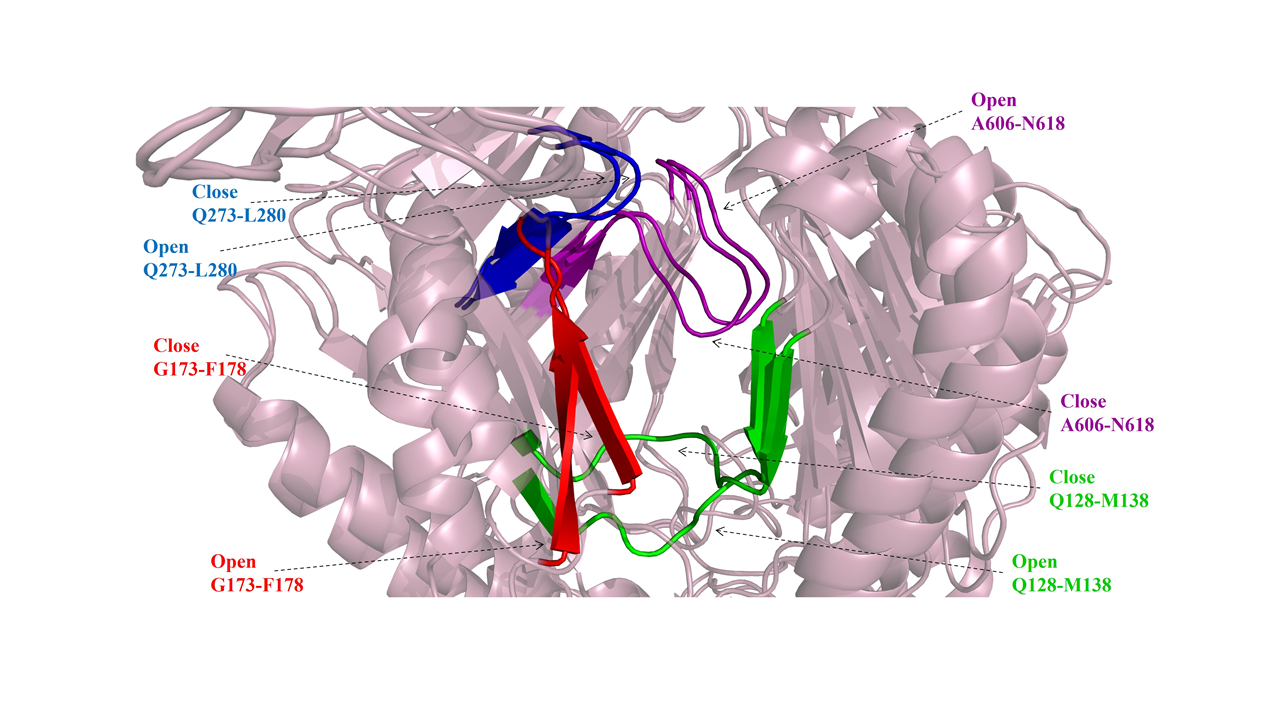

Supplement: FIG S7 [file msystems.00217-22-s0007.tif]
